# PD‐1 Inhibits CD4+ TRM‐Mediated cDC1 Mobilization via Suppressing JAML in Human NSCLC

**DOI:** 10.1002/advs.202507647

**Published:** 2026-01-04

**Authors:** Zheyu Shao, Qinyuan Liu, Zhongwei Xin, Zhiyao Zhou, Mingjie Lin, Di Chen, Zhixing Hao, Yongyuan Chen, Wenxuan Wu, Shuyang Zhang, Xiaoke Chen, Xia Xu, Jinfan Li, Wei Lin, Yuhao Lu, Dang Wu, Pin Wu

**Affiliations:** ^1^ Department of Thoracic Surgery The Second Affiliated Hospital Zhejiang University School of Medicine Zhejiang University Hangzhou China; ^2^ Key Laboratory of Tumor Microenvironment and Immune Therapy of Zhejiang Province The Second Affiliated Hospital Zhejiang University School of Medicine Zhejiang University Hangzhou China; ^3^ Department of Thoracic Surgery Shandong Provincial Hospital Affiliated to Shandong First Medical University Jinan China; ^4^ Department of Radiation Oncology The Second Affiliated Hospital Zhejiang University School of Medicine Zhejiang University Hangzhou China; ^5^ Department of Gastrointestinal Surgery The Second Affiliated Hospital Zhejiang University School of Medicine Hangzhou Zhejiang China; ^6^ Department of Orthopedic Surgery The Second Affiliated Hospital Zhejiang University School of Medicine Zhejiang University Hangzhou China; ^7^ Department of Pathology The Second Affiliated Hospital Zhejiang University School of Medicine Zhejiang University Hangzhou Zhejiang China; ^8^ Department of Thoracic Surgery The Second Affiliated Hospital Linping Campus Zhejiang University School of Medicine Zhejiang University Hangzhou China

**Keywords:** conventional type 1 dendritic cells (cDC1), junction adhesion molecule‐like protein (JAML), non‐small cell lung cancer (NSCLC), programmed cell death protein 1 (PD‐1), tissue‐resident memory CD4+ T cells (CD4+ TRMs), X‐C motif chemokine ligand 1 (XCL1)

## Abstract

Tissue‐resident memory CD4+ T cells (CD4+ TRMs) are pivotal in immune responses during inflammation and infection, yet their phenotype and function within the tumor microenvironment (TME) remain elusive. Here, we delineated CD4+ TRMs in non‐small cell lung cancer (NSCLC) using CD103 and CD69 as defining markers and demonstrated that their transcriptional and phenotypic profiles closely resembled those observed in murine models. Tumor‐infiltrating CD4+ TRMs exert helper antitumor effects by secreting the chemokine XCL1 to recruit conventional type 1 dendritic cells (cDC1s), facilitating antigen presentation and priming cytotoxic T lymphocyte responses. Mechanistically, we identified the costimulatory molecule JAML as essential for CD4+ TRM‐mediated cDC1 mobilization. Compared with their counterparts in normal lung tissue, tumor‐infiltrating CD4+ TRMs exhibited elevated expression of immune checkpoint molecules, indicating a dysfunctional state, accompanied by significantly reduced XCL1 expression. PD‐1 signaling within the NSCLC TME suppressed JAML expression—an effect reversible by PD‐1 blockade—while the administration of a JAML agonist further enhanced the antitumor efficacy of PD‐1 inhibitors in tumor‐bearing mice. Clinically, the presence of XCL1‐secreting CD4+ TRMs positively correlated with favorable clinical outcomes and enhanced responses to anti‐PD‐1 immunotherapy in NSCLC patients. Our findings reveal a critical role for JAML in facilitating CD4+ TRM‐mediated cDC1 mobilization within the NSCLC TME and highlight the translational potential of targeting CD4+ TRMs to enhance the efficacy of immune checkpoint blockade therapies.

## Graphical abstract

CD4+ tissue‐resident memory T cells (TRMs) in non‐small cell lung cancer recruit conventional type 1 dendritic cells via XCL1‐XCR1 signaling, orchestrating antitumor immunity. The costimulatory molecule JAML is essential for this process. PD‐1 blockade restores JAML expression and cDC1 mobilization, while JAML agonists synergize with anti‐PD‐1 therapy, offering promising immunotherapeutic strategies for NSCLC.

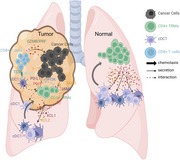

## 1 Introduction

Non‐small cell lung cancer (NSCLC) remains a leading cause of cancer‐related death globally, and despite immunotherapeutic advances, durable responses are achieved only in a subset of patients. A deeper understanding of the local immune landscape is therefore critical for improving clinical outcomes. In the lung—a site of constant exposure to pathogens and environmental stimuli [1, 2]—tissue‐resident memory T cells (TRMs) are uniquely positioned to mediate rapid and localized immune responses, contributing critically to both barrier protection and disease control [3]. Among the tissue‐resident immune populations, while CD8+ TRMs have already been linked to favorable prognosis and response to therapy in several solid tumors [4, 5], the functional relevance and phenotypic definition of CD4+ TRMs, particularly in human NSCLC, remain poorly defined.

Tissue‐resident memory CD4+ T cells (CD4+ TRMs) represent a specialized lineage of CD4+ memory T cells that are uniquely adapted to persist in peripheral tissues, including the skin [6], lung [1, 7], liver [8], gastrointestinal tract [9], reproductive tract [10, 11], lymphoid tissues [12] and brain [13]. Compelling evidence underscores the central role of CD4+ TRMs in mediating robust antiviral [11, 14] and antibacterial [15, 16, 17, 18] responses through the orchestrated mobilization of immune defenses. Nevertheless, paradoxically, CD4+ TRMs have also been shown to drive pathogenic effects in conditions such as systemic lupus erythematosus [6], ANCA‐associated glomerulonephritis [19], inflammatory bowel disease [20, 21, 22, 23], *Helicobacter pylori*‐positive gastritis [15] and allergic asthma [24, 25]. This duality highlights their plasticity in polarizing toward protective or pathogenic phenotypes, shaped by microenvironmental cues that guide their activation and differentiation [26]. Recent investigations in murine models of melanoma and breast cancer have revealed the ability of CD4+ TRMs to mediate anti‐tumor immunity [27]. With emerging evidence associating CD4+ TRMs with prognosis across multiple cancers [28, 29, 30], clarifying their functional role in human NSCLC is essential, especially considering the crucial influence of tissue‐resident immunity on immunotherapy effectiveness.

A major barrier to such understanding lies in the lack of consensus on the phenotypic criteria for human CD4+ TRMs. Foundational murine studies, which rely on parabiosis experiments [31, 32], have defined TRMs on the basis of canonical residency markers such as CD69 and CD103 [12, 32, 33, 34, 35], combined with a CD44^high^CD62L^low^ phenotype [36, 37]. However, translating these criteria to human systems is challenging [38], given the absence of parabiosis models, resulting in diverse and often conflicting marker definitions across tissues and disease contexts [6, 8, 9 ^,^ 11, 12, 16, 19, 39, 40, 41, 42]. In healthy human lung tissue, CD69+CD103+ CD4+ T cells are typically used to define CD4+ TRMs [7], whereas in inflammatory conditions—such as pneumococcal pneumonia [36, 43], pulmonary fibrosis [44], tuberculosis [45], and SARS‐CoV‐2 infection [46]—both CD69+ and/or CD103+ populations are variably considered. Such inconsistencies make it difficult to interpret the biological and clinical roles of CD4+ TRMs in lung cancer.

In this study, through integrated transcriptomic and proteomic analyses, we identified a distinct population of tumor‐infiltrating CD4+CD69+CD103+ tissue‐resident memory T cells (TRMs) within the human non‐small cell lung cancer (NSCLC). This population exhibited robust expression of canonical tissue‐residency signatures concomitant with diminished expression of tissue egress markers. Our investigation revealed for the first time that tumor‐infiltrating CD4+ TRMs actively recruit conventional type 1 dendritic cells (cDC1s) into the tumor microenvironment (TME) via the XCL1‐XCR1 signaling axis. Elevated XCL1 expression in CD4+ TRMs correlated significantly with improved clinical outcomes. Mechanistic analyses identified the co‐stimulatory molecule Junction Adhesion Molecule‐Like (JAML) as a critical regulator governing XCL1 secretion and consequent cDC1 recruitment by CD4+ TRMs. The PD‐1 signaling cascade, via PI3K pathway inhibition, attenuated JAML expression in tumor‐infiltrating CD4+ TRMs, thereby compromising their cDC1 recruitment capacity. Furthermore, anti‐PD‐1 treatment restored both JAML expression and cDC1 mobilization by CD4+ TRMs, and the combined administration of anti‐PD‐1 and JAML agonists synergistically inhibited tumor progression and prolonged survival in tumor‐bearing murine models. The abundance of tumor‐infiltrating CD4+ TRMs demonstrated positive correlation with anti‐PD‐1 immunotherapy efficacy in patients with NSCLC. Collectively, our findings elucidate the phenotypic and functional characteristics of tumor‐infiltrating CD4+ TRMs in human NSCLC, establishing their central role in orchestrating anti‐tumor immunity while elucidating the molecular mechanisms underlying their functional compromise within the immunosuppressive tumor microenvironment. These insights provide a compelling framework for developing innovative immunotherapeutic strategies targeting CD4+ TRMs and establishing robust prognostic biomarkers in human NSCLC.

## 2 Results

### 2.1 Co‐Expression of CD103 and CD69 Identifies CD4+ TRMs in Human NSCLC

**FIGURE 1:**
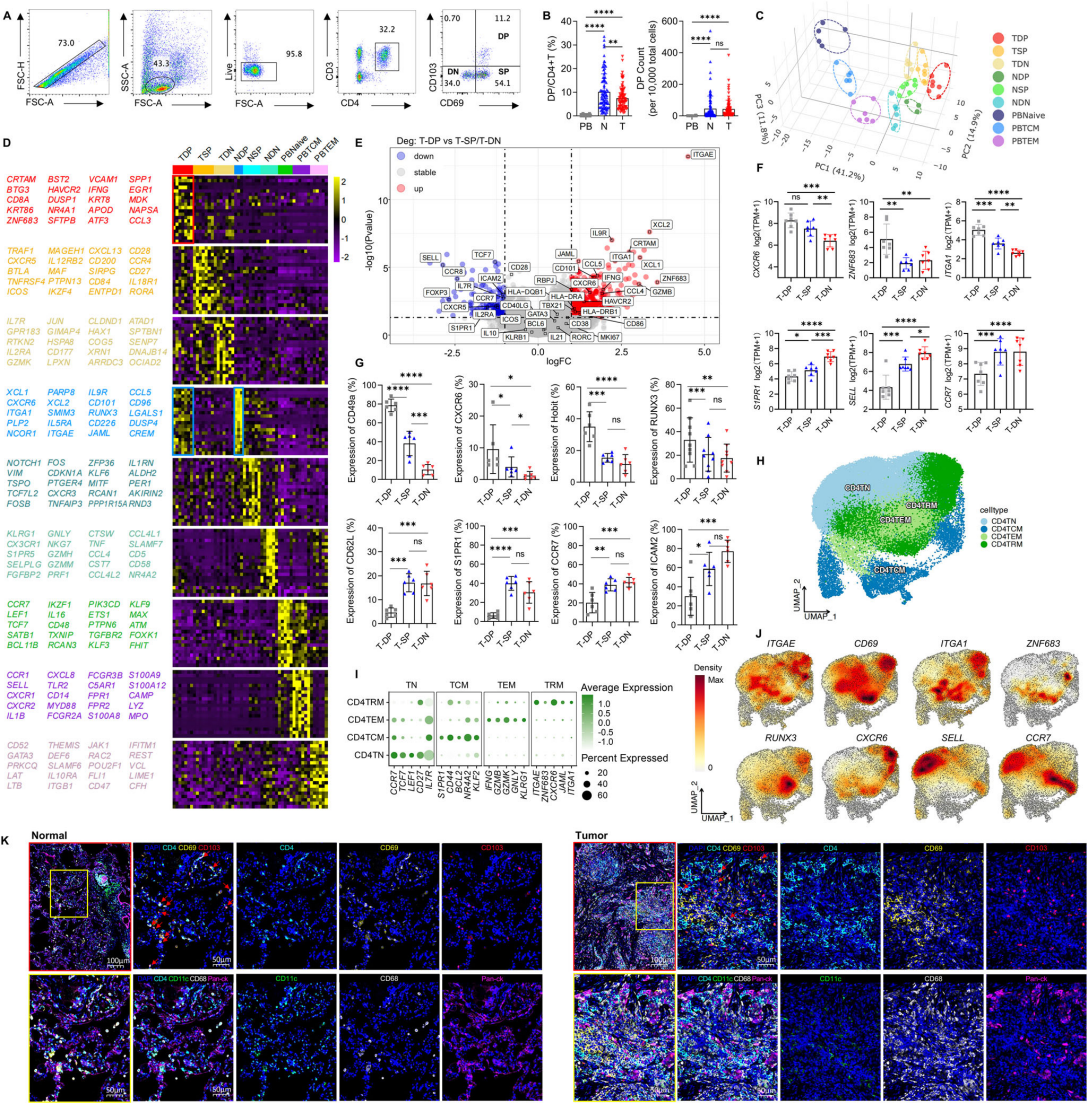
The identification of tumor‐infiltrating CD4+ TRMs in TME of human NSCLC. (A) Representative FCM gating strategy for CD4+ TRM identification in NSCLC tumors. Cells were sequentially gated on singlets, lymphocytes, live cells, and CD3^+^CD4^+^ T cells, followed by subdivision into CD69+CD103+ double positive (DP), CD69+CD103− single‐positive (SP), and CD69−CD103− double negative (DN) subsets. The percentages of the corresponding populations are indicated. (B) Frequencies and absolute counts of CD4+CD69+CD103+ TRMs (DP) among CD4+ T cells in peripheral blood (PB), normal lung tissue (N), and tumor tissue (T) from patients with NSCLC. Left, percentage of DP among total CD4^+^ T cells. Right, DP counts normalized per 10 000 total cells. (*n* = 92, paired). (C) 3d principal component analysis (3D PCA) of Bulk RNA‐seq data from nine subpopulations of CD4^+^ T cells in peripheral blood (PBNaive, PBTCM, PBTEM), normal tissue (NDP, NSP, NDN) and tumor tissue (TDP, TSP, TDN). N, normal tissue; T, tumor; PB, peripheral blood. TDP (*n* = 7), TSP (*n* = 7), TDN (*n* = 7), NDP (*n* = 3), NSP (*n* = 6), NDN (*n* = 6), PB Naive (*n* = 5), PB TEM (*n* = 6), and PB TCM (*n* = 6). (D) Heatmap showing the normalized average expression of the top 20 differentially expressed genes (DEGs) for each CD4+ T‐cell subset from peripheral blood (PB), normal lung tissue (N), and tumor tissue (T), as determined by bulk RNA‐seq. Each column represents an individual patient sample. Highly variable genes were identified by differential upregulated expression analysis across subsets. Representative marker genes are shown on the left. (E) Volcano plots showing the differentially expressed genes (DEGs) between TDP and Non‐TDP (TSP/TDN) subsets. DEGs were defined as P value < 0.05 and |LogFC| > 1. (F) Bar plots showing the log_2_(TPM+1) values of tissue residency‐related genes (*CXCR6*, *ZNF683*, *ITGA1*, *S1PR1*, *SELL*, and *CCR7*) in TDP, TSP, and TDN subsets. Measured by Bulk‐RNAseq. (G) The FCM analysis of tissue residency‐related markers (CD49a, *n* = 6; CXCR6, *n* = 6; Hobit, *n* = 6; RUNX3, *n* = 9; CD62L, *n* = 6; S1PR1, *n* = 6; ICAM2, *n* = 6; CCR7, *n* = 6) in the TDP, TSP, and TDN subsets. (H) UMAP visualization of single‐cell transcriptomes showing distinct CD4+ T‐cell subsets, including TN, TCM, TEM, and TRM. (I) Dot plot summarizing the expression of TRM‐associated genes across CD4+ T‐cell subsets. (J) Feature plots showing the distribution and expression density of representative TRM‐related markers (*ITGAE, CD69, ITGA1, ZNF683, RUNX3, CXCR6, SELL, and CCR7*). (K) Representative multiplex immunofluorescence images of normal lung tissue (left) and NSCLC tumor tissue (right) (Sample4). CD4 (cyan), CD69 (yellow), CD103 (red), CD11c (green), CD68 (white), Pan‐CK (magenta), nuclei (DAPI, blue) and merged images are shown. Insets show magnified views, with red arrows indicating the colocalization of CD4+CD69+CD103+ TRMs within the tissue microenvironment. Scale bars, 100 µM (overview) and 50 µM (magnified views). The data in (B, F, G) are shown as the mean ± SD. One‐way ANOVA with Tukey's post hoc test was used for statistical analysis; ns, non‐significant; *p < 0.05; **p < 0.01; ***p < 0.001; ****p < 0.0001.

Multiple markers have been utilized to define human CD4+ tissue‐resident memory T cells (TRMs), with CD103 and CD69 being the most prevalent [26]. In this study, we identified three major populations of CD4+ T cells in both tumor and paired lung tissues from patients with non‐small cell lung cancer (NSCLC): CD69+CD103+ (double positive, DP), CD69+CD103− (single positive, SP), and CD69−CD103− (double negative, DN), whereas CD69−CD103+ cells were essentially absent (Figure 1A; Extended Data Figure S1A). Of note, circulating CD4+ T cells in peripheral blood were almost exclusively DN, while all three subsets were present in both tumors and paired normal lungs, with distinct compartmental distributions (Figure 1B; Extended Data Figure S1B,C). To further characterize the transcriptional landscape, we sorted DP, SP, and DN cells from tumor and paired normal lung tissues, as well as naïve (TN), central memory (TCM), and effector memory (TEM) cells from the peripheral blood of NSCLC patients (matched patients for each cell subset) for Bulk RNA‐seq (Extended Data Figure S1D–F and Table S2). Analysis of 24,237 genes revealed that tumor‐infiltrating DP (TDP) cells formed a transcriptionally distinct cluster according to principal component analysis (PCA) (Figure 1C; Extended Data Figure S2A).

Heatmap analysis revealed high expression of canonical tissue‐residency genes in both TDP and NDP cells, with TDP cells showing relative downregulation compared with NDP, while TDP cells aberrantly expressed epithelial‐associated markers such as *KRT8*, *KRT86*, *SFTPB*, *NAPSA*, *APOD*, and *SPP1*, consistent with a T cell‐epithelium interaction signature [47, 48] (Figure 1D). TDP cells also specifically expressed *VCAM‐1*, *IFNG*, and *CRTAM2* (Figure 1D), molecules involved in the activation of CD4+ T cells [49] and promoting the differentiation of the CD4+ cytotoxic T lymphocyte (CTL) lineage [50]. Differential gene expression analysis highlighted that compared with TSP and TDN cells, TDP cells were enriched for *IL9R, XCL1, XCL2, CCL5, CCL4, JAML, IFNG, GZMB*, and *HAVCR2* but downregulated for *ICAM2, TCF7, RBPJ*, and *IL2RA* (Figure 1E; Extended Data Figure S2B,C). In addition, the expression of MHC class II‐related genes, including *HLA‐DRA, HLA‐DRB1*, and *HLA‐DQB1*, was significantly upregulated in TDP cells (Figure 1E; Extended Data Figure S2B,C). In contrast, the expression of Th2, Th17, Treg (regulatory T cells), and Tfh (T follicular helper cells)‐related genes, including *BCL6, RORC, GATA3, and TBX21*, did not significantly differ (Figure 1E; Extended Data Figure S2B,C). Statistical analysis further revealed that TDP cells preferentially expressed residency‐associated genes (*CXCR6, ZNF683, ITGA1*) while downregulating egress‐related genes (*S1PR1, SELL, CCR7*), in line with the core transcriptional features of TRM cells in both murine and human tissues [26] (Figure 1F). FCM analysis consistently confirmed the high expression of CD49a, CXCR6, Hobit, and RUNX3, and low expression of CD62L, S1PR1, CCR7, and ICAM2 in TDP cells (Figure 1G; Extended Data Figure S4A).

To further substantiate the identification of CD4+ TRMs in human lung cancer, we analyzed an integrated scRNA‐seq cohort of human NSCLC comprising 39 normal and 107 tumor samples (Extended Data Figure S2D). UMAP visualization resolved CD4+ T‐cell subsets, among which CD4+ TRMs were characterized by high *ITGAE, CD69, ITGA1, ZNF683*, and *CXCR6* expression and reduced *SELL* and *CCR7* expression (Figure 1H–J; Extended Data Figure S2E,F). Functionally, these cells were enriched for Th1/Tfh‐associated transcripts (e.g., *IFNG, RUNX3, TBX21, BCL6, and CXCR5*) and displayed minimal Treg signatures (e.g., *FOXP3*) (Extended Data Figure S2G). Correlation analysis further confirmed the coherence of the CD4+ TRM transcriptional program (Extended Data Figure S2H). FCM validation further refined their phenotypic features. T‐DP displayed a checkpoint‐high phenotype, with the highest PD‐1 and TIM‐3 and increased CTLA‐4 expression, whereas TIGIT expression did not consistently (Extended Data Figure S3A). The expression of the canonical costimulatory molecules CD28 and ICOS was only moderate, whereas that of the noncanonical molecule CD226 [51] was selectively upregulated in T‐DP cells (Extended Data Figure S3B). T‐DP also produced higher levels of the cytotoxic molecules IFN‐γ, granzyme B, and perforin, and resulted in the highest Ki‐67 expression, indicating a proliferative cytotoxic phenotype (Extended Data Figure S3C,D). Moreover, T‐DP cells presented reduced FOXP3 and CXCR5 but increased BCL6 expression (Extended Data Figure S3E). They also displayed elevated TOX with diminished TCF1 expression, consistent with terminal exhaustion (Extended Data Figure S3F). Additional tissue‐resident markers, including 2B4 and CCR5, were also specifically elevated in T‐DP cells (Extended Data Figure S3G).

To validate these findings in situ and assess spatial organization, we performed multiplex immunofluorescence (Figure 1K; Extended Data Figure S5). N‐DP cells were more abundant and formed compact clusters in normal lung tissues, whereas T‐DP cells were fewer, more dispersed, and located in closer proximity to tumor epithelial cells, suggesting tighter restraint by the tumor epithelium.

Previous studies have employed parabiosis [52, 53] and fate‐mapping [54] approaches to functionally validate the tissue residency of these cells in vivo [31, 32]. Here, we employed both intravenous CD45 labeling and FTY720‐mediated circulation blockade in a CMT‐167 orthotopic tumor model (Extended Data Figure S6A,F). The ivCD45 staining revealed that the majority of CD4+CD69+CD103+ DP cells in the lung and tumor remained unlabeled, indicating exclusion from the vascular compartment (Extended Data Figure S6B–E). Consistently, treatment with the S1P receptor antagonist FTY720 markedly depleted circulating CD4+ T cells but had little effect on DP cells within lung and tumor tissues, confirming their bona fide tissue‐resident identity (Extended Data Figure S6G–I).

On the basis of these comprehensive in vivo and in vitro multidimensional analyses, we established the CD4+CD103+CD69+ double‐positive (TDP) subset as bona fide tumor‐infiltrating CD4+ TRMs in human NSCLC, defined by canonical tissue‐resident memory features and distinct functional properties, consistent with findings across diverse tissues [19, 55, 56, 57, 58, 59] in both murine and human models.

### 2.2 Tumor‐Infiltrating CD4+ TRMs Play a Crucial Role in cDC1 Mobilization

**FIGURE 2:**
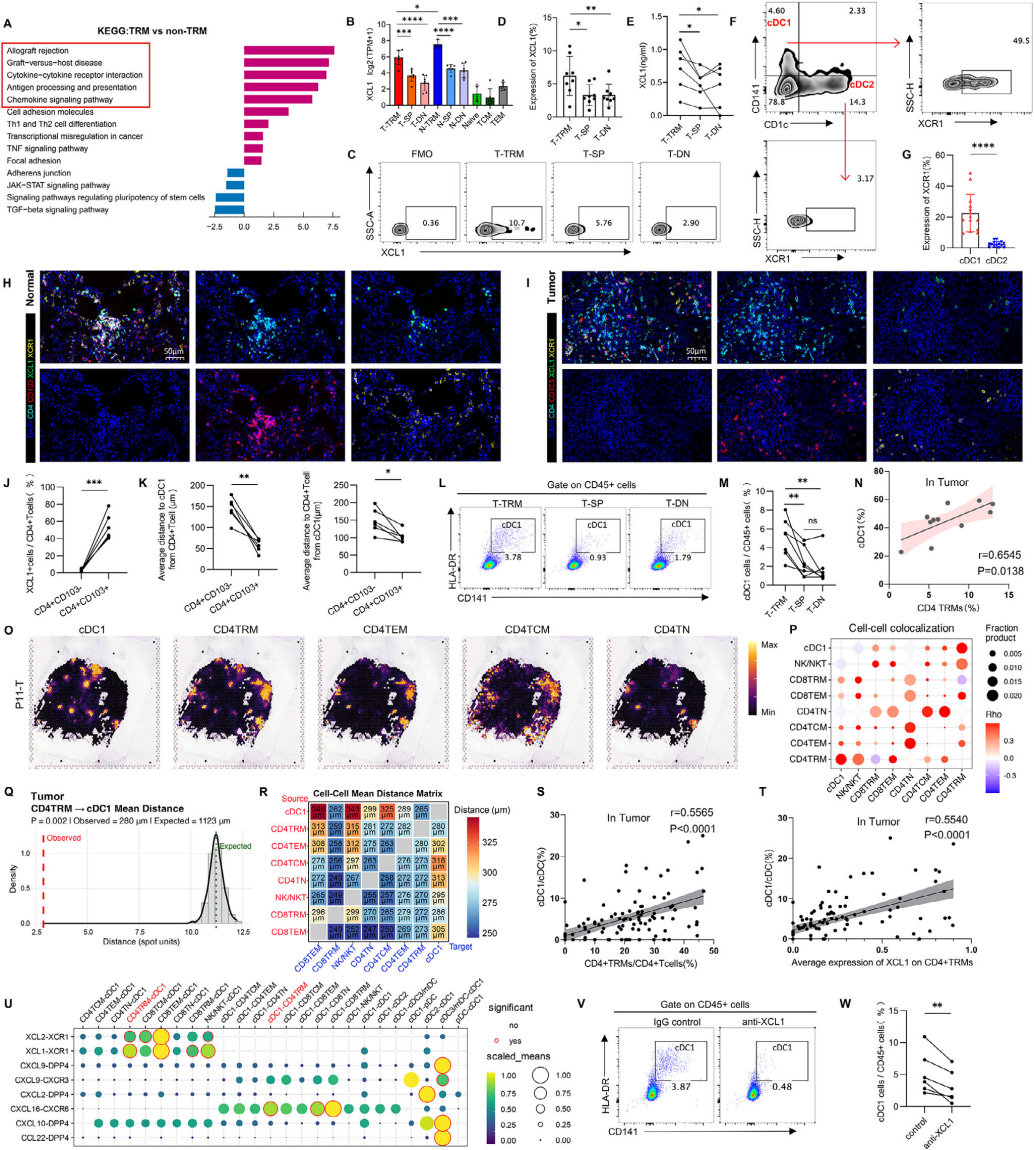
Tumor‐infiltrating CD4+ TRMs could recruit cDC1 toward TME. (A) KEGG pathway enrichment analysis comparing the TRMs and non‐TRM subsets. P value<0.05. (B) Bar plots depicting the log_2_(TPM+1) of *XCL1* expression across CD4+ T‐cell subpopulations, as determined by Bulk RNA‐seq. (C) Representative FCM plots showing XCL1 staining in the T‐TRM, T‐SP, and T‐DN subsets. The fluorescence minus one (FMO) control is shown on the left. The percentages indicate XCL1+ cells within each subset. (D,E) Quantitative analyses of XCL1 expression in T‐TRM, TSP, and TDN cells by FCM (*n* = 8; D) and ELISA (*n* = 6; E). Data from each matched pair are connected by folded lines. (F,G) Gating strategy for the FCM analysis of the XCR1 expression in cDC1 and cDC2 in tumor tissues. Bar graph depicting the frequency of XCR1 expression in the cDC1 and cDC2 populations. *n* = 14. (H,I) Representative multiplex immunofluorescence images of normal lung tissue (H) and NSCLC tumor tissue (I) (Sample1) showing the colocalization of CD4 (cyan), CD103 (red), XCL1 (green), XCR1 (yellow), nuclei (DAPI, blue), and the merged images. Scale bars, 50 µM. (J,K) Multiplex immunofluorescence quantification. (J) Frequencies of XCL1+ cells among CD4+CD103+ and CD4+CD103− T cells. (K) Nearest‐neighbor distances (µM): left, CD4+ T cell to the nearest cDC1; right, cDC1 to the nearest CD4+ T cell; both comparisons of CD4+CD103− with CD4+CD103+ subsets. The paired lines denote matched samples. *n* = 6. (L,M) Chemotaxis assay of cDC1 recruitment by tumor‐infiltrating CD4+ T‐cell subsets (*n* = 7). (L) Representative FCM plots showing cDC1 among all cells in the lower chamber after migration toward T‐TRM, T‐SP, or T‐DN groups. (M) Quantification of the cDC1 frequency among live cells in the lower chamber, comparing the three CD4+ T‐cell subsets (*n* = 7). (N) FCM analysis revealing the Spearman correlation between cDC1 frequency and CD4+ TRM frequency in tumor tissues (*n* = 11). r, Spearman correlation coefficient; p, p value. (O) Representative spatial transcriptomic maps (Sample P11‐T) showing the density distribution of cDC1, CD4TRM, CD4TEM, CD4TCM, and CD4TN across the section. (P) Cell‒cell colocalization dot plot showing spatial associations between cDC1, NK/NKT cells, CD8+ T‐cell subsets, and CD4+ T‐cell subsets. (Q) Distribution of observed versus expected mean distances from CD4TRMs to cDC1s in tumor tissues, showing a significantly shorter observed distance (280 µm) compared with expected (1123 µM). (R) Cell‒cell mean distance matrix across all tumor samples. Distances (µm) were calculated from each source cell type (rows) to target cell types (columns). (S,T) Spearman correlation analysis of the scRNA‐seq data in tumor samples (*n* = 107). (S) cDC1 proportions among total cDCs versus CD4+ TRM proportions among CD4+ T cells. (T) cDC1 proportion among total cDCs versus average XCL1 expression in CD4+ TRMs. r, Spearman correlation coefficient; p, p value. (U) Cell‒cell interaction dot plot showing ligand‒receptor pairs between cDC1 and T‐cell subsets. Ligands are shown on the left, and source–target cell pairs are indicated on the top. Red circles denote significant interactions. (V) Representative FCM plots gated on CD45+ cells showing the cDC1 frequency in the lower chamber after chemotaxis with IgG control (left) or anti‐XCL1 blockade (right). (W) Quantification of the cDC1 proportion among CD45+ cells in the lower chamber for each condition (*n* = 6). The data in (B, D, G) are expressed as the mean ± SD. Statistical analyses were performed using paired Student's t tests (G, J, K, W) and one‐way ANOVA with Tukey's post hoc test (B‐E, M); ns, non‐significant; *p < 0.05; ** *p* < 0.01; *** *p* < 0.001; **** *p* < 0.0001.

To elucidate the immunobiological role of tumor‐infiltrating CD4+ TRMs in lung cancer, we conducted an integrative functional enrichment analysis. Gene Ontology (GO) and Kyoto Encyclopedia of Genes and Genomes (KEGG) pathway analyses revealed robust enrichment in chemokine signaling, cytotoxic regulation, cell adhesion, and Th1/Th2 differentiation (Figure 2A; Extended Data Figure S7A). Previous studies have implicated XCL1 as a key chemokine for recruiting CD141+ conventional type 1 dendritic cells (cDC1) [60], with XCR1 being uniquely expressed on these cells, underscoring the central role of XCL1 in cDC1 mobilization [61]. Our Bulk RNA‐seq data further confirmed that both XCL1 and its homolog XCL2 were markedly overexpressed in tumor‐infiltrating CD4+ TRMs (Figures 1E and 2B; Extended Data Figure S7B), prompting us to investigate whether these CD4+ TRMs could actively recruit cDC1.

To test this hypothesis, we used FCM and enzyme‐linked immunosorbent assay (ELISA) to demonstrate that compared with other CD4+ T‐cell subsets, tumor‐infiltrating CD4+ TRMs secreted significantly higher levels of XCL1 (Figure 2C–E). We further confirmed that XCR1 was predominantly expressed on cDC1 and virtually absent on cDC2 by FCM (Figure 2F,G; Extended Data Figure S7C). The co‐localization of CD4, CD103, and XCL1 in both lung cancer and normal lung tissues was confirmed by multiplex immunofluorescence (Figure 2H,I; Extended Data Figure S7D). Quantitative analysis showed that CD4+CD103+ TRMs secreted XCL1 at significantly higher frequencies than CD4+CD103− T cells (Figure 2J). Spatial mapping further revealed that compared with their CD4+CD103− counterparts, CD4+CD103+ TRMs were positioned in closer proximity to cDC1 (Figure 2K). Together, these findings demonstrated that in the NSCLC tumor microenvironment (TME), the XCL1‐secreting function of CD4+ TRMs was closely coupled with their spatial association to cDC1. Corroborating these findings, transwell migration assays of cDC1 toward T cells revealed that compared to other CD4+ T cell subsets, tumor‐infiltrating CD4+ TRMs exhibited a significantly enhanced ability to recruit cDC1 (Figure 2L,M; Extended Data Figure S7E,F). FCM analysis revealed a positive correlation between the abundance of tumor‐infiltrating CD4+ TRMs and cDC1 within paired tissue samples, substantiating the chemotactic interaction (Figure 2N).

To further elucidate the functional crosstalk between CD4+ TRMs and cDC1, we performed cell2location‐based spatial deconvolution to annotate dendritic cell and T‐cell subsets in Visium spatial transcriptomic data from lung cancer (Figure 2O; Extended Data Figure S7G,H). This analysis revealed that CD4+ TRMs exhibited significantly higher co‐localization frequencies with cDC1 than other CD4+ T‐cell subsets (Figure 2O,P). Spatial distance quantification further showed that CD4+ TRMs were positioned closer to cDC1 than expected by random distribution, with a proximity ranking second only to that of CD8+ TRMs among NK/T‐cell subsets (Figure 2Q,R). In support of these findings, scRNA‐seq analysis confirmed a positive correlation between the abundance of tumor‐infiltrating CD4+ TRMs, XCL1 expression, and cDC1 infiltration (Figure 2S,T). Consistently, cell‒cell communication analysis identified cDC1 as the second most strongly interacting subset with CD4+ TRMs, following cDC2 (Extended Data Figure S7I). Moreover, among the T‐cell subsets, cDC1 displayed strong connections with CD4+ TRMs, CD8+ TRMs, and NK/NKT cells (Extended Data Figure S7I). Ligand‒receptor analysis identified the XCL1‐XCR1 axis as the key molecular pair driving the spatial association of CD4+ TRMs with cDC1 (Figure 2U). In addition, cDC1 were found to signal back to CD4+ TRMs through CXCL16‐CXCR6, suggesting a potential role in reinforcing their tissue residency [62, 63, 64]. Functionally, chemotactic assays conclusively demonstrated that the recruitment of cDC1 by CD4+ TRMs was XCL1 dependent (Figure 2V,W). Together, these findings provided the first comprehensive evidence that tumor‐infiltrating CD4+ TRMs actively recruited cDC1 via XCL1 secretion, uncovering a pivotal mechanism critical for dendritic cell mobilization and the orchestration of immune surveillance within the TME.

### 2.3 JAML Promotes the cDC1 Mobilization of CD4+ TRMs through XCL1 Secretion

**FIGURE 3:**
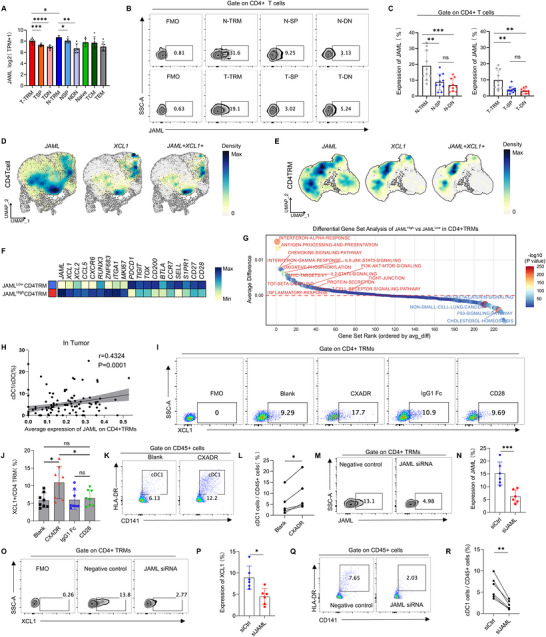
The co‐stimulatory JAML‐CXADR signaling promotes the recruitment of cDC1 by CD4+ TRMs. (A) Bar plots depicting the log_2_(TPM+1) of *JAML* expression across CD4+ T‐cell subpopulations, as determined by Bulk RNA‐seq. (B,C) JAML expression in CD4+ T‐cell subsets from paired normal lung and tumor tissue (*n* = 10). (B) Representative FCM plots gated on CD4+ T cells with FMO control, TRM, SP, and DN cells. The percentages indicate JAML+ cells. (C) Quantitative analyses of JAML expression across subsets in normal tissue (left) and tumor tissue (right). (D) UMAP visualization of the co‐localization density of *JAML, XCL1*, and *JAML+XCL1+* co‐expression density across all tumor‐infiltrating CD4+ T cells. (E) UMAP visualization of *JAML, XCL1*, and *JAML+XCL1+* co‐expression density across all tumor‐infiltrating CD4+ TRMs. (F) Heatmap showing the mean log‐normalized expression (Z score‐standardized) of chemokines, residency markers, inhibitory and co‐stimulatory molecules, and migration‐related genes in tumor‐infiltrating CD4+ TRMs, stratified by JAML^High^ vs. JAML^Low^ (P value<0.05). (G) Gene set enrichment analysis comparing JAML^High^ vs. JAML^Low^ CD4+ TRMs. Pathways are ranked by average expression difference, with the color indicating the –log_10_ (P) value. Red and blue labels indicate significantly upregulated and downregulated pathways, respectively. (H) Spearman correlation from scRNA‐seq in tumor samples (*n* = 107): cDC1 proportion among total cDCs versus average JAML expression in CD4+ TRMs. r, Spearman correlation coefficient; p, p value. (I,J) FCM analysis of XCL1 expression in tumor‐infiltrating CD4+ TRMs under four conditions: Blank, recombinant CXADR, IgG1 Fc and CD28 stimulation. (I) Representative plots. The FMO control is shown on the left. (J) Quantification of XCL1+ TRMs (*n* = 8). (K,L) Chemotaxis assay for cDC1 recruitment. (K) Representative FCM plots gated on CD45+ cells in the lower chamber after migration toward T‐TRM treated with Blank or CXADR stimulation. (L) Quantification of the cDC1 frequency among live CD45+ cells in the lower chamber (*n* = 5). (M,N) FCM analysis of JAML expression in tumor CD4+ TRMs after transfection with negative control siRNA (siCtrl) or JAML‐targeting siRNA (siJAML). (M) Representative plots. (N) Quantification of JAML+ CD4+ TRMs (*n* = 6). (O,P) FCM analysis of XCL1 expression in CD4+ TRMs following siRNA transfection. O, Representative plots gated on CD4+ TRMs (FMO, siCtrl, and siJAML). (P) quantification of XCL1+ CD4+ TRMs (*n* = 6). (Q,R) Chemotaxis assay of cDC1 recruitment after coculture with CD4+ TRMs transfected with siCtrl or siJAML. (Q) Representative plots gated on CD45+ cells showing the frequency of cDC1. (R) quantification of cDC1 among CD45+ cells in the lower chamber (*n* = 6). The data in (A, C, J, N, and P) are expressed as the mean ± SD. Statistical analyses were performed using paired Student's t tests (L, N, P, and R) and one‐way ANOVA with Tukey's post hoc test (A, C, J). ns, non‐significant; *p < 0.05; ** *p* < 0.01; *** *p* < 0.001; **** *p* < 0.0001.

Junctional adhesion molecule‐like protein (JAML), also known as adhesion molecule interacting with CXADR antigen 1 (AMICA1), plays a pivotal role in modulating anti‐tumor immunity and immune cell migration [65]. Growing evidence supports that JAML could activate CD8+ T cells, CD4+ T cells, and γδ T cells, even at low concentrations [66]. Our Bulk RNA‐seq and flow cytometry analyses revealed that JAML expression was significantly elevated in CD4+ TRMs compared to other CD4+ T cell subsets in both tumor and normal tissues from patients with NSCLC (Figure 3A–C). Additionally, GeneMANIA analysis demonstrated that JAML‐CXADR interactions, along with their associated molecules, were involved in key cellular processes such as migration, adhesion, and chemotaxis (Extended Data Figure S8A). We further examined data from the TISIDB database, uncovering a positive correlation between JAML expression and the abundance of activated CD4+ T cells in lung adenocarcinoma (LUAD) and lung squamous cell carcinoma (LUSC) tumors (Extended Data Figure S8B). Similarly, analysis of TCGA data also revealed a significant positive correlation between JAML expression and CD4+ TRM signature expression in NSCLC (Extended Data Figure S8C). Consistent with these findings, our Bulk RNA‐seq analysis indicated a trend toward positive correlation between JAML and XCL1 expression in tumor‐infiltrating CD4+ TRMs (Extended Data Figure S8D).

We next investigated the upstream regulatory features of JAML in CD4+ TRMs by examining its transcriptional distribution across T‐cell subsets in NSCLC. UMAP projections revealed a strong overlap in distribution between JAML and XCL1 expression, both of which were enriched within the CD4+ T cells compartment (Figure 3D). Among all T‐cell subsets, the co‐expression of JAML and XCL1 was predominantly confined to TRMs (Extended Data Figure S8E,F). Within the TRM population, cells with higher JAML expression (JAML^high^) also exhibited increased *XCL1* expression (Figure 3E). Moreover, JAML^high^ CD4+ TRMs upregulated tissue residency‐associated genes (*CXCR6, RUNX3, ITGA1, and ZNF683*) while downregulating immunosuppressive molecules (*PDCD1, TIGIT, CD200, BTLA, and TOX*) and egress‐associated receptors (*S1PR1, SELL, and CCR7*) (Figure 3F). The *CD28* and *CD27* were more highly expressed in JAML^low^ TRMs (Figure 3F), consistent with a less differentiated state and tighter linkage to the circulating pool [67, 68]. Pathway enrichment analysis further demonstrated that JAML^high^ TRMs were enriched in interferon signaling, chemokine‐mediated migration, antigen presentation, inflammatory responses, and the TGF‐β and PI3K‐AKT pathways, which was consistent with an activated and immunoregulatory phenotype. In contrast, JAML^low^ TRMs were enriched for metabolic programs such as cholesterol biosynthesis and p53 signaling, reflecting a less activated state (Figure 3G). Supporting these findings, scRNA‐seq analysis revealed a significant positive correlation between the average *JAML* expression in TRMs and the cDC1 abundance within tumors (Figure 3H).

To further assess whether JAML functionally regulated XCL1 production and cDC1 recruitment, tumor‐infiltrating CD4+ TRMs were stimulated through the CXADR‐JAML pathway. Compared with CD28 costimulation, this activation markedly increased XCL1 production, as measured by intracellular FCM (Figure 3I,J). In parallel, chemotaxis assays revealed that CXADR engagement substantially increased the capacity of CD4+ TRMs to recruit cDC1 (Figure 3K,L). Moreover, siRNA‐mediated knockdown of JAML in CD4+ TRMs markedly reduced their ability to secrete XCL1 and recruit cDC1 (Figure 3M–R). In summary, our data established that the expression and activation of JAML were crucial for the secretion of XCL1 and the subsequent mobilization of cDC1 by tumor‐infiltrating CD4+ TRMs in human NSCLC. These findings highlight the critical role of JAML in modulating immune cell trafficking and may offer new insights into strategies aimed at enhancing anti‐tumor immunity through targeted modulation of JAML signaling pathways.

### 2.4 The Recruitment of cDC1 by CD4+ TRMs Was Suppressed in TME

Building on our finding that CD4+ TRMs mediate cDC1 recruitment through JAML‐dependent XCL1 secretion, to clarify how the TME reprogrammed TRM function, we next sought to compare the functional integrity of CD4+ TRMs within tumors (T‐TRM) with that of their counterparts in adjacent normal lung tissue (N‐TRM).

**FIGURE 4:**
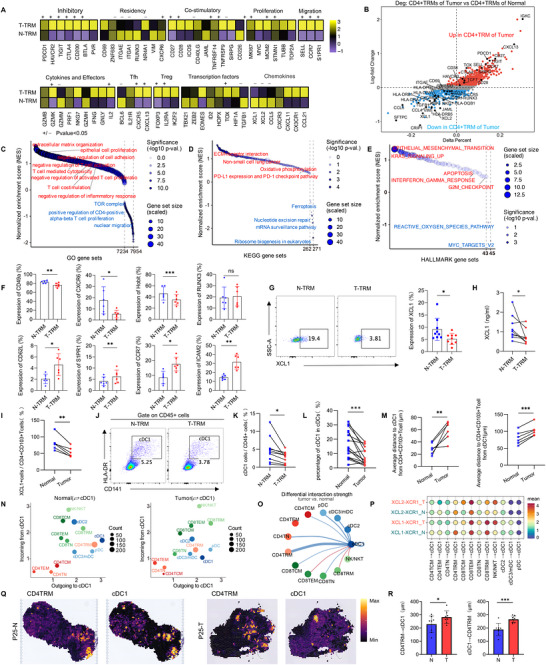
Functional Impairment of Tumor‐Infiltrating CD4+ TRMs. (A) Bulk RNA‐seq analysis revealing the heatmap of the mean log‐normalized (Z score‐standardized) expression of genes related to inhibition, tissue residency, costimulation, proliferation, migration, cytokines, Tfh‐associated genes, Treg‐associated genes, transcription factors and chemokines in the N‐TRM and T‐TRM. Differentially expressed genes (P value<0.05) are indicated by ‘+’ (upregulated) or ‘–’ (downregulated). (B) Volcano plot showing the results of the scRNA‐seq analysis of differentially expressed genes, plotted as the log fold change versus differences in the percentage of cells expressing each gene for TRMs in tumor versus normal tissues (adjusted P value<0.05, Wilcoxon rank‐sum test). (C–E) Gene set enrichment analysis (GSEA) of tumor versus normal CD4+ TRMs. (C) GO gene sets. (D) KEGG gene sets. (E) HALLMARK gene sets. Normalized enrichment scores (NESs) are shown. Red, gene sets enriched in tumor CD4+ TRMs; blue, gene sets enriched in normal CD4+ TRMs. (F) Bar plots showing the expression levels of tissue residency‐related genes (CD49a, CXCR6, Hobit, RUNX3, CD62L, S1PR1, CCR7, and ICAM2) between normal and tumor tissues using FCM analysis (*n* = 6 per group for all markers). (G) FCM analysis of XCL1 expression in CD4+ TRMs between normal (N‐TRM) and tumor (T‐TRM) tissues, with representative plots (left) and quantification (right) (*n* = 9). (H) Quantification of XCL1 expression (ng/mL) in N‐TRM and T‐TRM by ELISA (*n* = 9). (I) Multiplex immunofluorescence quantification of XCL1+ cells among CD4+ CD103+ T cells in paired normal and tumor tissues (*n* = 6). (J,K) Chemotaxis assay of cDC1 recruitment by CD4+ TRMs. (J) Representative FCM plots gated on CD45+ cells showing the cDC1 frequency in the N‐TRM group versus T‐TRM group. (K) Quantification of the cDC1 frequency among CD45+ cells in the lower chamber, comparing N‐TRM and T‐TRM (*n* = 10). (L) FCM analysis showing the percentages of cDC1 among cDCs between tumor and normal tissues (*n* = 16). (M) Multiplex immunofluorescence quantification of the spatial proximity between CD4+CD103+ T cells and cDC1 in normal and tumor tissues. Left, average distance from CD4+CD103+ T cells to the nearest cDC1; right, average distance from cDC1 to the nearest CD4+CD103+ T cell (*n* = 6). (N) Cell‒cell communication analysis of cDC1‐centric interactions in normal (left) and tumor (right) tissues. Bubble plots display outgoing signaling to cDC1 (x‐axis) and incoming signaling from cDC1 (y‐axis) across immune cell types, including CD4+ T cells, CD8+ T cells, NK/NKT cells, and other dendritic cell subsets. The bubble size reflects the interaction count, and the color denotes cell type classification. (O) Differential cDC1‐centered cell‒cell communication between tumor and normal tissues. Edges link cDC1 to partner immune subsets; edge width reflects the magnitude of change in aggregated interaction strength, and color indicates direction (red, higher in tumors; blue, higher in normal). (P) Dot‐heatmap depicting XCL1‐XCR1 and XCL2‐XCR1 signaling to cDC1 in tumor (T) and normal (N) tissues. Columns represent distinct ligand‐producing immune cell types (CD4+ T cells, CD8+ T cells, NK/NKT cells, and other dendritic cell subsets) engaging cDC1. (Q,R) Representative spatial transcriptomic analysis of CD4+ TRMs and cDC1 in normal (N) and tumor (T) tissues (Samples P25‐N and P25‐T). (Q) Spatial density maps showing the distribution of CD4+ TRMs and cDC1 across the tissue section. The color scale indicates local cell abundance. (R) Quantification of the mean nearest‐neighbor distances between CD4+ TRMs and cDC1 in both directions (*n* = 6). The data in (F–I, K–M, and R) are expressed as the mean ± SD. Statistical analyses were all performed using paired Student's t‐tests. ns, non‐significant; *p < 0.05; **p < 0.01; ***p < 0.001; ****p < 0.0001.

We first performed a comprehensive phenotypic and transcriptomic comparison. Integrated transcriptomic analyses combining Bulk and single‐cell RNA‐seq revealed that, compared with their normal lung counterparts, tumor‐infiltrating CD4+ TRMs exhibited significant upregulation of genes associated with immune checkpoint regulation (*PDCD1, HAVCR2, TIGIT, CTLA4, CD200*, and *BTLA4*), cell proliferation (*MYC, MCM2, TUBB*, and *TOP2A*), migratory capacity (*CCR7, SELL*, and *S1PR1*), co‐stimulatory signaling (*CD27, CD28, ICOS, TNFRSF9, and SIRPG*), and T cell exhaustion (*TOX)* (Figure 4A,B). Conversely, genes governing tissue residency tissue residency (*ITGAE, ITGA1, RUNX3, and VIM*), chemokine production (*XCL1, XCL2, CCL5*), noncanonical costimulation (*JAML*), and MHC class II antigen presentation (*HLA‐DPA1, HLA‐DPB1, HLA‐DQA1, and HLA‐DQB1*) showed profound downregulation within the tumor microenvironment (Figure 4A,B). Furthermore, we conducted enrichment analyses based on the GO, KEGG, and HALLMARK gene sets (Figure 4C–E). The results revealed that CD4+ TRMs in the TME were significantly enriched in tumor‐associated programs, including epithelial‐mesenchymal transition (EMT), KRAS signaling, interferon‐γ response, extracellular matrix remodeling, epithelial proliferation, oxidative phosphorylation, and PD‐1/PD‐L1 checkpoint signaling (Figure 4C–E). These findings suggested that tumor‐infiltrating CD4+ TRMs were confined to a suppressive niche shaped by stromal remodeling, metabolic reprogramming, and immune checkpoint activation, resulting in the acquisition of functional impairment and exhaustion. In contrast, CD4+ TRMs in normal lung tissue displayed a homeostatic profile marked by elevated anabolic activity, strong proliferative capacity, and long‐term survival potential (Figure 4C–E). Notably, the abundance of CD4+ TRMs was lower in tumors than in normal lung tissue (Figure 1B), and flow cytometry further confirmed the loss of tissue residency markers in tumor‐infiltrating CD4+ TRMs (Figure 4F; Extended Data Figure S9A). These data indicated that the TME drove a phenotypic shift in CD4+ TRMs from a stable resident state toward a dysfunctional, immunosuppressed phenotype.

We next focused on the alteration of the cDC1‐mobilizing function of tumor‐infiltrating CD4+ TRMs, which secreted significantly less XCL1 than their counterparts from matched normal lung tissues (Figures 2B and 4G,H), as further validated by multiplex immunofluorescence staining (Figures 2H,I and 4I). This deficit in XCL1 production was paralleled by a corresponding reduction in the recruitment of conventional type 1 dendritic cells (cDC1s) within the tumor microenvironment (TME) relative to that in normal lungs (Figure 4J,K). FCM analysis further confirmed that the cDC1 abundance was markedly diminished in tumors compared with paired normal tissues (Figure 4L). We next assessed cDC1 functionality in tumors versus normal lungs and reported that tumor‐derived cDC1s exhibited reduced expression of XCR1 together with lower levels of effector cytokines and activation markers (Extended Data Figure S9B–K). Moreover, multiplex immunofluorescence analysis demonstrated an increased separation between cDC1s and CD4+ TRMs in tumors compared with normal lung tissues (Figures 2H,I and 4M). To elucidate the crosstalk between CD4+ TRMs and cDC1s, cell‒cell communication analysis revealed strong outgoing signals from CD4+ TRMs to cDC1s in normal lungs, which were markedly attenuated in tumors (Figure 4N). Differential network analysis further demonstrated that the CD4+ TRM‐cDC1 axis was preferentially disrupted in the TME, with compensatory strengthening of alternative inputs from CD8+ TRMs, CD8+ TEMs, and NK/NKT cells (Figure 4O; Extended Data Figure S10A). Consistently, expression profiling of the XCL1‐XCR1 axis revealed a marked reduction of CD4+ TRM‐derived XCL1‐engaged cDC1s in tumors compared with normal lungs (Figure 4P). Spatial transcriptomics further confirmed the diminished co‐localization of CD4+ TRMs and cDC1s in tumors, contrasting with their close spatial coupling in adjacent normal tissues (Figure 4Q; Extended Data Figure S10B). Quantitative distance analysis validated these observations, revealing significantly increased separation between CD4+ TRMs and cDC1s in tumors (Figure 4R). Taken together, these results demonstrated that the NSCLC TME disrupted the XCL1‐XCR1 axis and spatial coordination between CD4+ TRMs and cDC1s, thereby impairing their recruitment and functional interplay essential for effective anti‐tumor immunity.

### 2.5 PD‐1 Attenuates the cDC1 Mobilization by Suppressing JAML Expression in Tumor‐Infiltrating CD4+ TRMs

**FIGURE 5:**
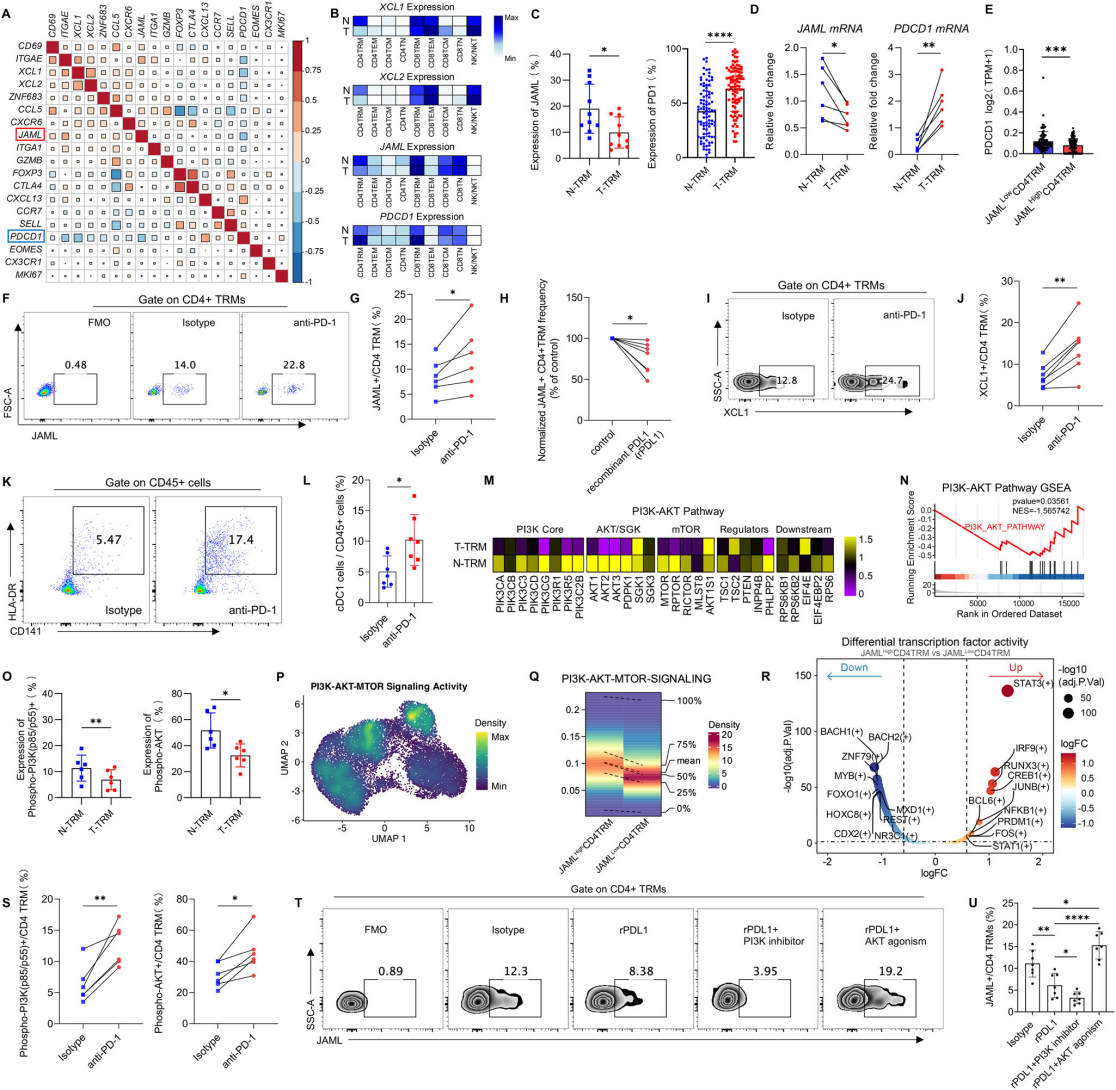
Immune checkpoint PD‐1 inhibits the cDC1 mobilization of CD4+ TRMs via reducing JAML expression. (A) Single‐cell RNA‐seq analysis showing a Spearman correlation matrix of TRM‐associated genes in tumor CD4+ TRMs. The colors indicate pairwise correlation coefficients (red: positive, blue: negative). (B) Heatmaps showing the expression of *XCL1, XCL2, JAML*, and *PDCD1* across CD4+ and CD8+ T‐cell subsets and NK/NKT cells in normal (N) and tumor (T) tissues. The color scale indicates relative expression (blue, high; white, low). (C) FCM analysis of JAML (*n* = 10) and PD1 (*n* = 94) expression in CD4+ TRMs from paired normal (N‐TRM) and tumor (T‐TRM) tissues. (D) qPCR analysis of *JAML* and *PDCD1* mRNA expression in sorted CD4+ TRMs from paired normal (N‐TRM) and tumor (T‐TRM) tissues (*n* = 6). (E) Single‐cell RNA‐seq analysis showing log_2_(TPM+1) values of *PDCD1* expression in JAML^Low^ and JAML^High^ CD4+ TRMs. (F,G) FCM analysis of JAML expression in CD4+ TRMs within tumor single‐cell suspensions following anti‐PD‐1 treatment. (F) Representative plots gated on CD4 TRMs (FMO, isotype, and anti‐PD‐1 groups). (G) Quantification of JAML+ CD4 TRMs comparing isotype versus anti‐PD‐1 treatment (*n* = 6). (H) FCM analysis of sorted tumor‐infiltrating CD4+ TRMs stimulated with recombinant PD‐L1 (rPDL1). The data were normalized to those of the control group and shown as the relative frequency of JAML+ CD4+ TRMs (*n* = 6). (I,J) FCM analysis of XCL1 production in CD4+ TRMs within tumor single‐cell suspensions following anti‐PD‐1 treatment. (I) Representative plots gated on CD4+ TRMs (isotype and anti‐PD‐1 groups). (J) Quantification of XCL1+ CD4+ TRMs comparing isotype versus anti‐PD‐1 treatment (*n* = 7). (K,L) Chemotaxis assay of cDC1 recruitment induced by CD4+ TRMs sorted from tumor single‐cell suspensions following anti‐PD‐1 treatment. (K) Representative FCM plots gated on CD45+ cells showing the cDC1 frequency in the lower chamber (isotype and anti‐PD‐1 groups). (L) Quantification of the cDC1 frequency among CD45+ cells in the isotype and anti‐PD‐1 groups (*n* = 7). (M) Bulk RNA‐seq analysis of the PI3K‐AKT signaling pathway in N‐TRM and T‐TRM. Heatmap showing the relative expression of PI3K core components, AKT/SGK kinases, mTOR pathway genes, upstream regulators, and downstream effectors. The color scale indicates normalized expression levels. (N) Bulk RNA‐seq gene set enrichment analysis (GSEA) of the PI3K‐AKT pathway in which T‐TRM were compared with N‐TRM. Negative enrichment (blue) indicates reduced pathway activity in T‐TRM relative to N‐TRM (NES = −1.57, P = 0.036). (O) FCM analysis of PI3K‐AKT pathway activation in CD4+ TRMs. The frequencies of phospho‐PI3K (p85/p55)+ (left) and phospho‐AKT+ (right) cells among N‐TRM and T‐TRM are shown(*n* = 6). (P) UMAP visualization of PI3K‐AKT‐mTOR signaling activity in tumor CD4+ TRMs derived from single‐cell GSVA analysis. (Q) Density plot of PI3K‐AKT‐mTOR signaling activity comparing JAML^High^ and JAML^Low^ CD4+ TRMs. (R) Volcano plot showing differential transcription factor activity between JAML^High^ and JAML^Low^ CD4+ TRMs. Red denotes transcription factors that were upregulated in JAML^High^ CD4+ TRMs, and blue denotes those that were upregulated in JAML^Low^ CD4+ TRMs. (S) FCM analysis of PI3K‐AKT pathway activation in CD4+ TRMs within tumor single‐cell suspensions following anti‐PD‐1 treatment. The frequencies of phospho‐PI3K (p85/p55)+ (left) and phospho‐AKT+ (right) cells in the isotype and anti‐PD‐1 groups are shown (*n* = 6). (T,U) FCM analysis of JAML expression in sorted CD4+ TRMs under different treatments. (T) Representative plots showing JAML+ CD4 TRMs in the FMO, isotype, rPDL1, rPDL1 plus PI3K inhibitor, and rPDL1 plus AKT agonist conditions. (U) Quantification of the frequency of JAML+ CD4+ TRM cells across treatment groups (*n* = 7). The data in (C, E, L, O, and U) are expressed as the mean ± SD. Statistical analyses were performed using paired Student's t‐tests (C‐E, G, H, J, L, O, and S) and one‐way ANOVA with Tukey's post hoc test (U). ns, non‐significant; *p < 0.05; **p < 0.01; ***p < 0.001; ****p < 0.0001.

The transition of CD4+ TRMs into a dysfunctional state in tumors prompted us to investigate upstream regulators of their reduced cDC1‐recruiting capacity. Among these genes, PD‐1 signaling stood out as a plausible suppressor of JAML expression [69]. Single‐cell RNA sequencing delineated an inverse correlation between *PDCD1* expression and the expression of residency‐associated markers (*CD69, ITGAE, ZNF683, and CXCR6*) as well as key chemokines (*XCL1, XCL2, and CCL5*) and the co‐stimulatory molecule *JAML* within tumor‐infiltrating CD4+ TRMs (Figure 5A). Consistently, comparative analysis across NK/T‐cell subsets between normal and tumor conditions revealed that the reduction expression of *JAML*, *XCL1*, and *XCL2* was restricted to CD4+ TRMs, whereas *PDCD1* expression was preferentially elevated in the same population, further underscoring a potential regulatory link between PD‐1 signaling and JAML (Figure 5B). Further reinforcing these observations, tumor‐infiltrating CD4+ TRMs displayed significantly higher PD‐1 expression concomitant with reduced levels of JAML when compared with CD4+ TRMs from normal lung tissue (Figure 5C,D). This dichotomy prompted the hypothesis that aberrant activation of the PD‐1/PD‐L1 pathway within the TME suppresses JAML expression in tumor‐infiltrating CD4+ TRMs.

To functionally validate this link, we first discovered that, in line with the above results (Figure 3F), JAML^low^ CD4+ TRMs expressed higher levels of *PDCD1* than their JAML^high^ counterparts (Figure 5E). Blockade of PD‐1 in tumor single‐cell suspensions upregulated JAML expression in CD4+ TRMs (Figure 5F,G), whereas the addition of recombinant PD‐L1 to purified tumor‐infiltrating CD4+ TRMs suppressed JAML expression (Figure 5H). To further validate the potential effect of anti‐PD‐1 treatment on the cDC1‐recruiting program, we showed that anti‐PD‐1 increased XCL1 in CD4+ TRMs within tumor single‐cell suspensions (Figure 5I,J) and, upon sorting after anti‐PD‐1 exposure, CD4+ TRMs exhibited enhanced cDC1‐recruiting function in a transwell assay (Figure 5K,L).

To elucidate the detailed mechanistic pathways involved in JAML modulation and PD‐1 signaling, we further examined the downstream components potentially regulated by the PD‐1 pathway. Through transcriptomic and FCM analyses, we found that PI3K‐AKT‐mTOR signaling was broadly attenuated in tumor‐infiltrating CD4+ TRMs (Figure 5M–O; Extended Data Figure S11A, B), consistent with their reduced JAML expression. UMAP visualization of GSEA scores for the PI3K pathway revealed a strong overlap in the distribution of pathway activity and JAML expression within CD4+ TRMs (Figure 5P). Moreover, JAML^high^ CD4+ TRMs exhibited stronger PI3K‐AKT‐mTOR activity (Figure 5Q), with increased activity of pathway‐associated transcription factors, including STAT3 [70, 71], STAT1 [72, 73], CREB1 and NFKB1 [74, 75], and reduced activity of FOXO1 [76] and BACH2 [77], factors negatively associated with this pathway (Figure 5R). Functionally, PD‐1 blockade restored PI3K‐AKT‐mTOR activity, as indicated by elevated levels of p‐PI3K and p‐AKT in CD4+ TRMs (Figure 5S; Extended Data Figure S11C,D). Causality was further established by perturbation, wherein recombinant PD‐L1 suppressed JAML expression, and PI3K inhibition further reduced JAML under PD‐L1 exposure, whereas AKT agonism rescued JAML expression (Figure 5T,U). Overall, these results demonstrated that PD‐1 signaling constrained the cDC1‐mobilizing capacity of tumor‐infiltrating CD4+ TRMs by repressing JAML expression through inhibition of the PI3K‐AKT‐mTOR pathway.

### 2.6 PD‐1 Blockade Promotes JAML Signaling to Enhance CD4+ TRM‐Mediated Anti‐Tumor Immunity

**FIGURE 6:**
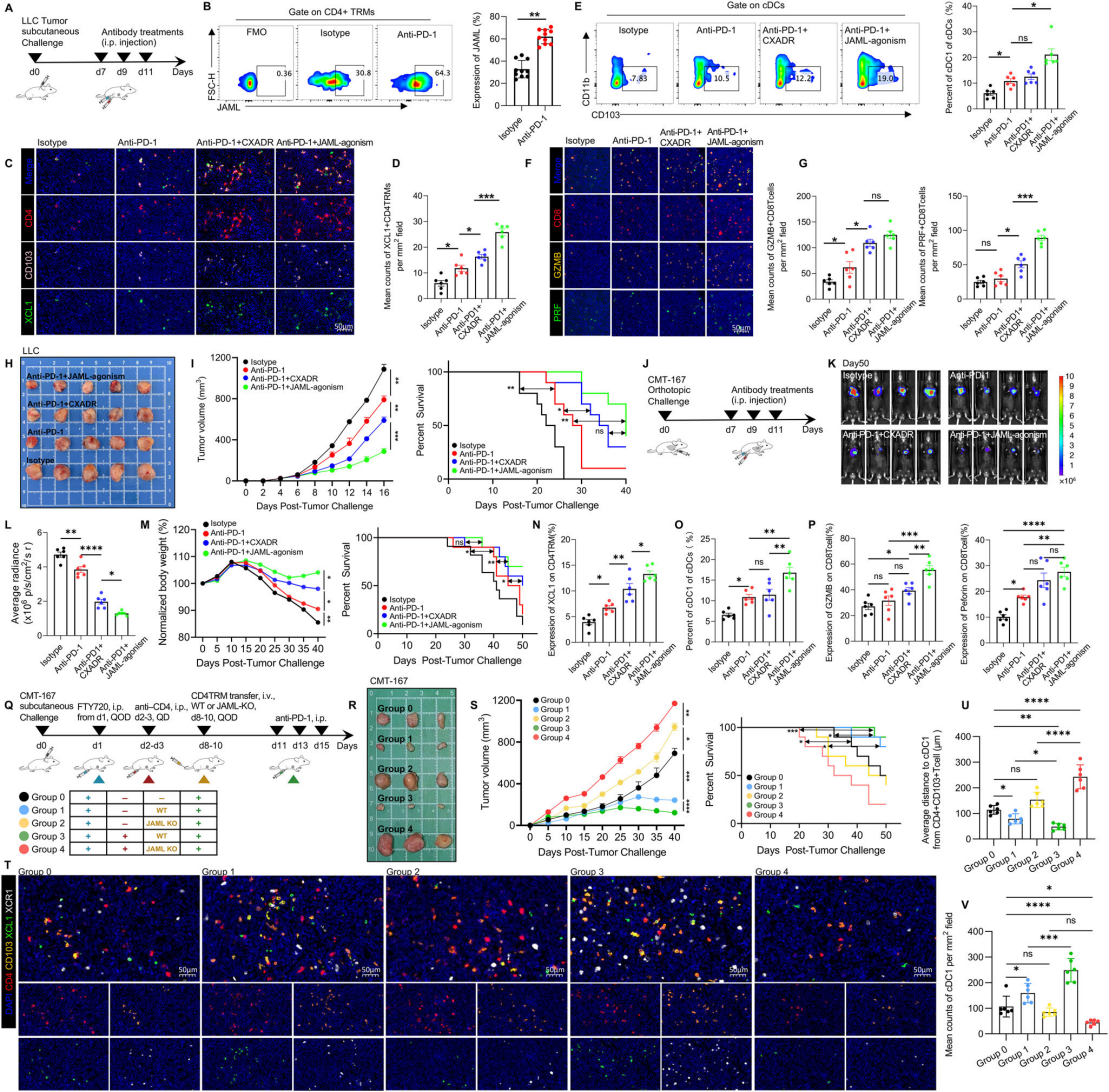
JAML‐agonism in combination with PD‐1 blockade enhances CD4+ TRM‐mediated anti‐tumor immunity. (A) Experimental timeline: C57BL/6 mice were subcutaneously implanted with LLC cells and received i.p. injections of isotype IgG, anti‐PD‐1, anti‐PD‐1+recombinant CXADR, or anti‐PD‐1+JAML‐agonism on Days 7, 9, and 11 after tumor challenge. (B) FCM analysis of JAML expression in CD4+ TRMs. Left, representative plots (FMO, isotype, anti‐PD‐1) gated on CD4+ TRMs; right, quantification of JAML+ CD4+ TRMs (*n* = 10). (C,D) Multiplex immunofluorescence of LLC tumors across treatment groups (isotype, anti‐PD‐1, anti‐PD‐1+CXADR, and anti‐PD‐1+JAML‐agonism groups). (C) Representative images showing CD4 (red), CD103 (pink), XCL1 (green), nuclei (DAPI, blue) and merged images. Scale bar, 50 µm. (D) Quantification of XCL1+ CD4+ TRMs per mm^2^ field (*n* = 6). (E) FCM analysis of cDC1 within cDCs across treatment groups. Left, representative plots gated on cDCs showing the cDC1 frequency for isotype, anti‐PD‐1, anti‐PD‐1+CXADR, and anti‐PD‐1+JAML‐agonism groups; right, quantification of cDC1 among cDCs (*n* = 6). (F,G) Multiplex immunofluorescence of LLC tumors across treatment groups (isotype, anti‐PD‐1, anti‐PD‐1+CXADR, and anti‐PD‐1+JAML‐agonism). (F) Representative images showing CD8 (red), GZMB (yellow), PRF (green), nuclei (DAPI, blue), and merged images. Scale bar, 50 µM. (G) Quantification of GZMB+ CD8 T cells (left) and PRF+ CD8 T cells (right) per mm^2^ field (*n* = 6). (H) Macroscopic images of subcutaneous LLC tumors from mice treated with isotype, anti‐PD‐1, anti‐PD‐1+CXADR and anti‐PD‐1+ JAML‐agonism antibodies. (I) Tumor growth curves (left) and Kaplan‒Meier survival curves (right) for LLC‐bearing C57BL/6 mice among the four groups (*n* = 10). (J) Experimental timeline: C57BL/6 mice were orthotopically implanted with CMT‐167 cells and received i.p. injections of isotype IgG, anti‐PD‐1, anti‐PD‐1+recombinant CXADR, or anti‐PD‐1+JAML‐agonism antibodies on Days 7, 9, and 11 after tumor challenge. (K) In vivo IVIS Spectrum imaging of CMT‐167 orthotopic tumor‐bearing mice in the supine position at Day 50 post‐treatment with isotype IgG, anti‐PD‐1, anti‐PD‐1+CXADR, or anti‐PD‐1+JAML‐agonism antibodies. (L) Quantification of tumor bioluminescence by IVIS on Day 50, showing the average radiance (×10^6^ p/s/cm^2^/sr) in the isotype, anti‐PD‐1, anti‐PD‐1+CXADR, and anti‐PD‐1+JAML‐agonism groups (*n* = 6). (M) Body weight and survival of CMT‐167 orthotopic tumor‐bearing mice. Left, Normalized body weight (%) over time in isotype, anti‐PD‐1, anti‐PD‐1+CXADR, and anti‐PD‐1+JAML‐agonism groups. Right, Kaplan‒Meier survival curves of the four treatment groups (*n* = 10). (N–P) FCM quantification of immune subsets in the CMT‐167 orthotopic model across treatment groups (isotype, anti‐PD‐1, anti‐PD‐1+CXADR, and anti‐PD‐1+JAML‐agonism groups). (N) Expression of XCL1 on CD4+ TRMs. (O) Percentage of cDC1 among cDCs. (P) Expression of GZMB (left) and perforin (right) on CD8+ T cells (*n* = 6). (Q) Experimental timeline: C57BL/6 mice were subcutaneously implanted with CMT‐167 cells and received i.p. injections of FTY720 (from Day 1, QOD), anti‐CD4 antibody (on Days 2–3, QD), adoptive transfer of WT or JAML‐KO CD4 TRMs (on days 8–10, QOD), and anti‐PD‐1 antibody (on Days 11, 13, and 15) after tumor challenge. Group assignment: All groups received FTY720 and anti‐PD‐1 antibodies. Group 0: control; Group 1: only WT CD4+ TRM transfer; Group 2: only JAML‐KO CD4+ TRM transfer; Group 3: anti‐CD4 plus WT CD4+ TRM transfer; Group 4: anti‐CD4 plus JAML‐KO CD4+ TRM transfer. (R) Macroscopic images of CMT‐167 tumors from mice in Groups 0–4, as defined in the experimental design. (S) Tumor growth curves (left) and Kaplan‒Meier survival analysis (right) of CMT167‐bearing mice across Group 0–4 (*n* = 10). (T) Representative multiplex immunofluorescence images of CMT‐167 tumors from Groups 0–4, showing CD4 (red), CD103 (yellow), XCL1 (green), XCR1 (white), nuclei (DAPI, blue), and merged images. Scale bars, 50 µm. (U) Average distance from CD4+CD103+ T cells to the nearest cDC1 (µM) in CMT‐167 tumors across Group 0–4. (V) Mean counts of cDC1 per mm^2^ field in CMT‐167 tumors across Group 0–4. Scale bar, 50 µM. The data in (B, D, E, G, L, N–P, and U,V) are expressed as the mean ±SD. Statistical analyses were performed using unpaired Student's t‐tests (B) and one‐way ANOVA with Tukey's post hoc test (D, E, G, L, N–P, and U,V). ns, non‐significant; *p < 0.05; **p < 0.01; ***p < 0.001; ****p < 0.0001.

Having established the mechanistic basis of impaired cDC1 mobilization by CD4+ TRMs, we next evaluated the functional relevance of targeting this regulatory axis in vivo. In Lewis lung carcinoma (LLC) subcutaneous tumor model (Figure 6A), PD‐1 blockade consistently significantly restored JAML expression in CD4+ TRMs within tumors (Figure 6B; Extended Data Figure S12A). Multiplex immunofluorescence further confirmed the increase in XCL1 secretion, which was further amplified upon concomitant activation of the JAML‐CXADR axis (Figure 6C,D; Extended Data Figure S12H). Similarly, cDC1 infiltration within tumors was significantly increased, with the strongest effect observed under combined PD‐1 blockade and JAML agonism, whereas PD‐1 blockade alone or in combination with recombinant CXADR exerted only a limited effect (Figure 6E; Extended Data Figure S12B). Functionally, cDC1 mobilization was accompanied by an increase in cytotoxic CD8+ T cells expressing granzyme B and perforin [78], with the strongest effect observed under the combinatorial regimen (Figure 6F,G; Extended Data Figure S12I). In line with these immunological changes, PD‐1 blockade combined with JAML agonism reduced tumor volume by an additional 31.4% over PD‐1 monotherapy and significantly prolonged survival (Figure 6H–J). Considering that the therapeutic benefit conferred by the JAML agonistic antibody was not reproduced well by recombinant CXADR, likely due to tumor progression‐associated downregulation of CXADR, which limited its efficacy (Extended Data Figure S12C,D). A similar expression pattern was observed in melanoma models, in which PD‐1 blockade also failed to restore CXADR levels [69]. To validate these observations in a more physiological lung tumor conditions, we next evaluated the therapeutic effects in an orthotopic CMT167‐luc model (Figure 6J). Consistent with the LLC findings, combined PD‐1 blockade and JAML agonism resulted in superior tumor control and survival benefits (Figure 6K–M). Notably, the differences among treatment groups were more pronounced in this orthotopic setting, with mice receiving the combination therapy displaying not only higher survival rates than those in the LLC model but also progressive tumor regression at later stages and partial recovery of body weight, underscoring the heightened sensitivity to PD‐1 blockade (Figure 6M). FCM analyses also revealed that with escalating treatment efficacy, CD4+ TRMs exhibited enhanced XCL1 production (Figure 6N; Extended Data Figure S12E), accompanied by robust recruitment of cDC1 (Figure 6O; Extended Data Figure S12E) and marked expansion of cytotoxic CD8+ T cells expressing granzyme B and perforin (Figure 6P; Extended Data Figure S12E).

We further confirmed the essential role of CD4+ TRMs and JAML signaling in mediating anti‐tumor effects in the context of PD‐1 blockade by performing adoptive transfer experiments in a CMT‐167 subcutaneous model, intravenously transferring CD4+ TRMs isolated from the lungs of C57 mice, either wild‐type (WT) or JAML‐deficient (JAML KO) (Figure 6Q; Extended Data Figure S12F). To rigorously assess the contribution of tumor‐infiltrating TRMs, all the mice were treated with FTY720 to block lymphocyte egress, and in selected groups, transient anti‐CD4 antibody administration was used to deplete endogenous CD4+ T cells prior to transfer (Extended Data Figure S12G). Tumor growth and survival analyses revealed a clear therapeutic gradient across the five groups (Figure 6R,S). The most pronounced benefit was observed in Group 3, where transient depletion of endogenous CD4+ T cells followed by WT TRMs transfer maximized the effect of PD‐1 blockade (Figure 6R,S). Compared with controls (Group 0), mice receiving WT TRMs without depletion (Group 1) also showed significant improvement, but the effect was less striking, likely reflecting residual suppression from endogenous CD4+ T cells, particularly Tregs (Figure 6R,S). These findings further suggested that among CD4+ T‐cell subsets, JAML‐expressing CD4+ TRMs constituted the principal responders to PD‐1 blockade, whereas other CD4+ T cells contributed little to the therapeutic benefit and might even dilute the efficacy. In contrast, the introduction of JAML‐deficient TRMs not only failed to provide protection (Group 2) but also further impaired the therapeutic outcome when combined with CD4 depletion (Group 4), underscoring the indispensable role of JAML in sustaining TRM‐mediated tumor control (Figure 6R, S). Moreover, multiplex immunofluorescence (Figure 6T; Extended Data Figure S12J) demonstrated that the therapeutic benefit was accompanied by tighter spatial coupling of CD4+ TRMs with cDC1s (Figure 6U; Extended Data Figure S12K) and a significant increase in intratumoral cDC1 density (Figure 6V), whereas these effects were markedly attenuated following the transfer of JAML‐deficient TRMs.

Together, these findings unequivocally established CD4+ TRMs and JAML signaling as central mediators of cDC1 recruitment and anti‐tumor immunity under PD‐1 blockade, and highlighted a combinatorial strategy whereby PD‐1 inhibition coupled with JAML agonism synergistically augmented therapeutic efficacy, charting a promising avenue for next‐generation immunotherapy.

### 2.7 Tumor‐Infiltrating CD4+ TRMs Correlate with Prognosis and Immunotherapy Efficacy in Human NSCLC

**FIGURE 7:**
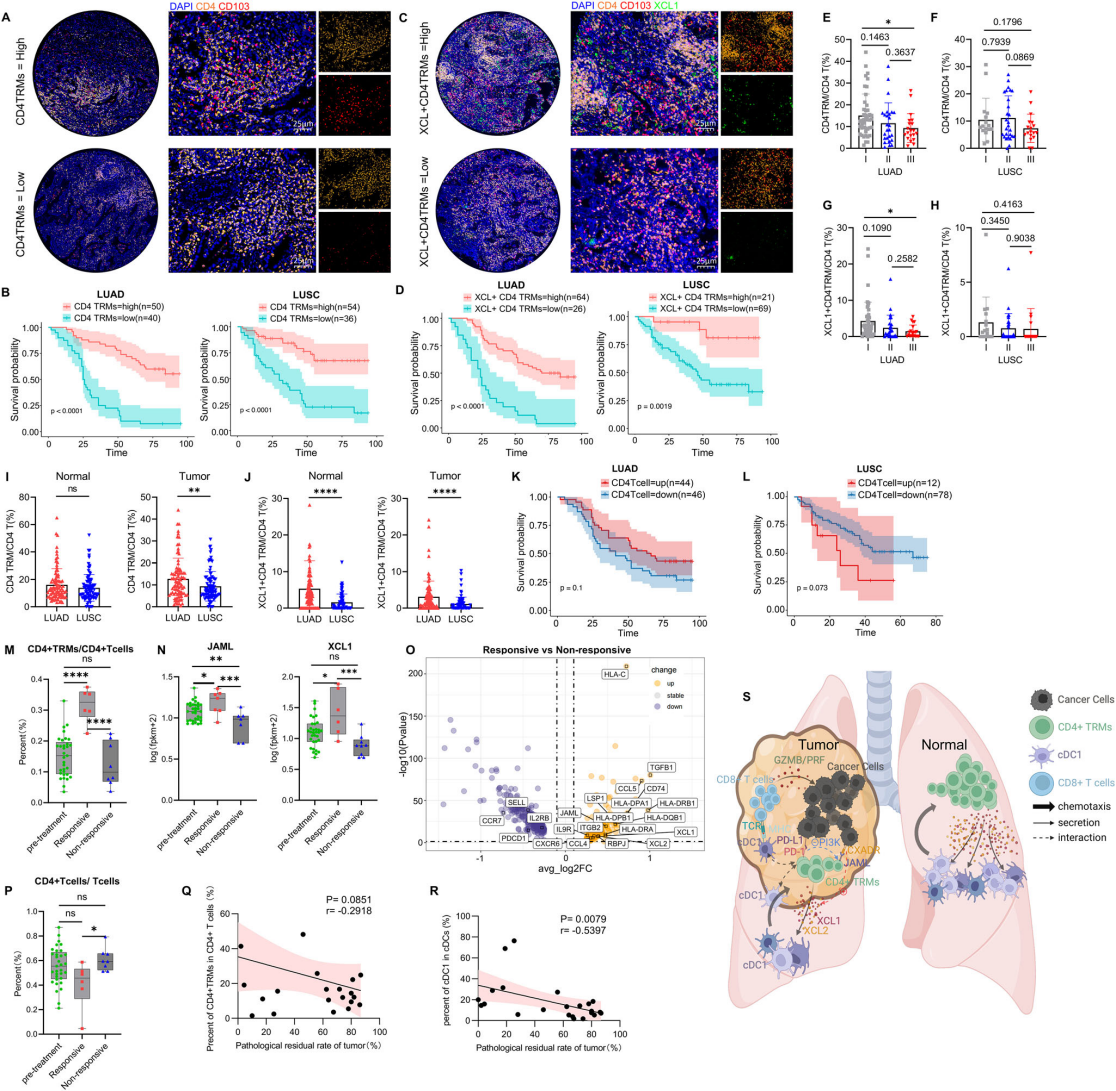
Tumor‐infiltrating CD4+ TRMs correlate with patient prognosis and immunotherapy efficacy. (A) Representative IF staining images showing the localization of CD4 (orange), CD103 (red), and DAPI (nuclei, blue) in the tissue microarray. Scale bars, 25 µM. (B) Kaplan‒Meier overall survival (OS) curves stratified by the percentage of CD4+ TRMs in lung adenocarcinoma (LUAD, *n* = 90) (left) and lung squamous cell carcinoma (LUSC, *n* = 90) (right). High and low groups were defined using optimal cutoff values, and differences in OS were assessed using the log‐rank test. (C) Representative IF staining images showing the localization of CD4 (orange), CD103 (red), XCL1 (green), and DAPI (nuclei, blue) in the tissue microarray. Scale bars, 25 µm. (D) Kaplan‒Meier OS curves based on the percentage of XCL1+ CD4+ TRMs in LUAD (*n* = 90, left) and LUSC (*n* = 90, right). Optimal cutoff values were used to define high versus low groups, and statistical comparisons were performed using the log‐rank test. (E,F) Bar graphs showing the proportions of CD4+ TRMs among total CD4+ T cells across TNM stages in LUAD (E, *n* = 44, 26, and 20 for stages I, II, and III, respectively) and LUSC (F, *n* = 16, 27, and 18 for stages I, II, and III, respectively). (G,H) Bar graphs showing the frequency of XCL1+ CD4+ TRMs among CD4+ T cells across TNM stages in LUAD (G, *n* = 44, 26, and 20 for stages I, II, and III, respectively) and LUSC (H, *n* = 16, 27, and 18 for stages I, II, and III, respectively). (I,J) Bar graphs comparing the proportions of CD4+ TRMs (I) and XCL1+ CD4+ TRMs (J) among total CD4+ T cells from paired normal (left) and tumor tissues (right) from LUAD (*n* = 90) and LUSC (*n* = 90). (K,L) Kaplan‒Meier OS curves based on intratumoral CD4+ T‐cell abundance in LUAD (*n* = 90, left) and LUSC (*n* = 90, right). Optimal cutoff values were used to define high versus low groups, and statistical comparisons were performed using the log‐rank test. (M,N) Proportions of CD4+ TRMs among total CD4+ T cells (M) and the log_2_(fpkm+1) of *JAML* and *XCL1* expression (N) in pre‐treatment, responsive, and non‐responsive groups. (O) Volcano plots presenting the DEGs between the responsive groups and non‐responsive groups. P value < 0.05, |LogFC| > 0.1. (P) Frequencies of CD4+ T cells among total T cells in pre‐treatment, responsive, and non‐responsive groups. (Q,R) Correlation analyses showing the relationship between CD4+ TRMs within CD4+ T cells (Q) or cDC1 within cDCs (R) and the pathological residual tumor rate following anti‐PD‐1 immunotherapy (*n* = 23). Spearman correlation coefficients (r) and P values are indicated. (S) Schematic model illustrating how tumor‐infiltrating CD4+ TRMs secrete XCL1 to recruit cDC1, which in turn promote CD8+ T‐cell‐mediated anti‐tumor responses; note that cDC1 recruitment is inhibited within the tumor microenvironment. This schematic was generated using BioRender (https://www.biorender.com). The data in (E–H, I,J, M,N, and P) are expressed as the mean ± SD. Statistical analyses were performed using unpaired Student's t‐tests (I, J) and one‐way ANOVA with Tukey's post hoc test (E–H, M, N, and P). ns, non‐significant; *p < 0.05; **p < 0.01; ***p < 0.001; ****p < 0.0001.

Understanding the prognostic impact of CD4+ TRMs in human NSCLC is critical, and we therefore analyzed their abundance and function in relation to patient survival. IF staining of clinical LUAD and LUSC cohorts (Extended Data Figure S13A and Tables S5 and S6) revealed marked inter‐patient heterogeneity in the abundance of CD4+ TRMs, consistent with the highly individualized immune landscapes of tumors (Figure 7A). Patients with higher densities of tumor‐infiltrating CD4+ TRMs, stratified by optimal cutoff values determined using maximally selected rank statistics, exhibited prolonged overall survival (OS) and improved prognosis (Figure 7A,B; Extended Data Figure S13F,G). Interestingly, while the overall abundance of CD4+ TRMs was reduced in tumors compared with adjacent normal lungs (Extended Data Figure S13B,C), a subset of patients displayed higher intratumoral levels, which correlated with superior outcomes (Extended Data Figure S13D,E). In terms of functional phenotype, the abundance of XCL1+ CD4+ TRMs also varied markedly across patients and, based on optimal stratification, showed a significant positive correlation with OS in both LUAD and LUSC (Figure 7C,D; Extended Data Figure S13F,G). LUAD exhibited greater infiltration of CD4+ TRMs and functional XCL1+ subsets at baseline and in early tumor stages, but showed steeper losses during progression, positioning these cells as key immune variables driving prognostic differences and highlighting fundamental differences in immune dependency between LUAD and LUSC (Figure 7E–J). Importantly, the total CD4+ T cells abundance was not predictive of survival, highlighting that the prognostic value was restricted to the tissue‐resident phenotype rather than to bulk CD4+ T cells infiltration (Figure 7K,L; Extended Data Figure S13F,G). Consistently, data derived from the TCGA further demonstrated that gene signatures associated with both CD4+ TRMs and cDC1 were both closely linked to favorable survival outcomes in patients with NSCLC (Extended Data Figure S13H,I).

To evaluate the clinical relevance of these findings in the context of immune checkpoint therapy, we next analyzed scRNA‐seq data from NSCLC tumor biopsies obtained before and during PD‐1 blockade combined with chemotherapy, enabling the identification of CD4+ TRM subsets within tumors (Extended Data Figure S14A,B and Table S7). In responders, anti‐PD‐1 therapy induced a marked increase in CD4+ TRMs infiltration together with elevated expression of JAML and XCL1, all of which were consistently greater than those in nonresponders, where such induction was absent and JAML expression was even reduced (Figure 7M,N; Extended Data Figure S14C–E). Higher baseline abundance of CD4+ TRMs and elevated JAML and XCL1 expression appeared to indicate favorable therapeutic responses (Extended Data Figure S14C–E). Comprehensive transcriptomic analysis revealed that CD4+ TRMs in responsive tumors upregulated chemokines (*XCL1, XCL2, CCL5, and CCL4*), MHC class II genes (*HLA‐DPA1, HLA‐DPB1, HLA‐DRB1, and HLA‐DQB1*), and *JAML*, while concomitantly downregulating immune checkpoint and trafficking molecules (*PDCD1, CCR7, and SELL*), consistent with a more activated and less immunosuppressive phenotype (Figure 7O). Moreover, we noted that the overall abundance of CD4+ T cells was lower in responders, underscoring the selective prognostic and therapeutic value of the TRM compartment (Figure 7P; Extended Data Figure S14F). To further elucidate the immunotherapeutic landscape, mass cytometry of NSCLC tumors (Table S8) treated with neoadjuvant anti‐PD‐1 [79] revealed that both intratumoral CD4+ TRMs abundance and cDC1 density correlated inversely with residual tumor burden, wheras the overall CD4+ T cells proportion was associated with poor prognosis, highlighting a CD4+ TRM‐cDC1 axis specifically linked to therapeutic response (Figure 7Q,R; Extended Data Figure S15A–E). These findings highlighted the prognostic and predictive value of CD4+ TRMs and reinforced the central importance of the CD4+TRM‐JAML‐XCL1‐cDC1 axis in shaping responses to PD‐1 based immunotherapy.

In summary, we constructed a model whereby dysfunctional CD4+ TRMs in the NSCLC TME drive immune escape, and showed that their functional restoration through combined PD‐1 blockade and JAML‐CXADR agonism provides a compelling therapeutic strategy to augment anti‐tumor immunity and improve clinical outcomes (Figure 7S).

## 3 Discussion

CD4+ tissue‐resident memory T cells (TRMs) have been unequivocally detected across a wide array of human peripheral tissues and are emerging as pivotal regulators of immune homeostasis or, conversely, as key participants in various immune dysregulation‐related disorders [26]. However, within malignant tumors, their characterization remains largely elusive. In this study, we comprehensively explored the phenotypic, transcriptomic, and functional landscapes of tumor‐infiltrating CD4+ TRMs in human non‐small cell lung cancer (NSCLC). Our findings revealed that tumor‐resident CD4+ T cells co‐expressing CD103 and CD69 exhibited hallmark features consistent with those of previously reported CD4+ TRMs, thereby reaffirming their bona fide tissue‐resident identity [26]. These cells orchestrated the recruitment of conventional type 1 dendritic cells (cDC1) via secretion of the chemokine XCL1. We further reported a striking overexpression of the novel co‐stimulatory molecule JAML in these cells, with activation of the JAML pathway emerging as a critical regulator of XCL1 release. Our results revealed that within the NSCLC tumor microenvironment (TME), CD4+ TRMs acquired a dysfunctional phenotype characterized via a cascade of inhibitory signals—most prominently, an elevated PD‐1 expression that, through suppression of the PI3K signaling pathway, downregulated JAML expression and, consequently, impaired cDC1 mobilization. The abundance and functional status of these cells correlated with improved patient prognosis and responsiveness to immune checkpoint inhibitors (ICIs), while targeted activation of JAML appeared to enhance anti‐PD‐1 therapeutic efficacy.

The definition of CD4+ TRMs has long been a subject of debate [38]. Early murine studies relied on markers such as CD11a and CD69 [80], whereas subsequent studies shifted their focus to CD69 as a singular defining feature [81]. In contrast, initial human studies primarily utilized CD69 expression [33, 34], and more recent investigations have adopted the co‐expression of CD103 and CD69 to delineate CD4+ TRMs [9, 34, 82, 83, 84]. These discrepancies underscore species‐ and tissue‐specific variations in CD4+ TRMs phenotypes, highlighting the need for a refined and unified definition—especially in the context of disease. By leveraging these widely accepted markers, our study reinforced that CD103 and CD69 co‐expression constituted a robust strategy for identifying tumor‐infiltrating CD4+ TRMs in NSCLC, thereby laying the groundwork for deeper insights into their functional roles and therapeutic potential within the TME.

While the contributions of CD4+ TRMs to infectious disease immunity have been well documented, their function in tumor immunity remain less clearly delineated. Recent murine studies have implicated CD4+ TRMs in the initiation and maintenance of anti‐tumor responses in models of melanoma and breast cancer [27]. Clinically, the density of CD4+ TRMs has been linked to favorable patient outcomes in several malignancies [28, 29, 30]. Our data extend these findings by demonstrating that CD4+ TRMs are indispensable for conventional type 1 dendritic cells (cDC1) recruitment through the secretion of XCL1 [85]—a chemokine critical for the formation, spatial organization, and maintenance of tissue‐resident memory T cells [86]. This observation is in line with prior evidence of CD4+ TRMs facilitating macrophage mobilization during HSV‐2 infection [11]. It is interesting that we reported a role for CD8+ TRMs in cDC2 mobilization in another study [79], suggesting that both CD4+ and CD8+ TRMs synergistically facilitate antigen cross‐presentation in human NSCLC. Specifically, CD4+ TRMs appear to amplify local antigen presentation and potentiate cytotoxic T‐cell priming, thereby fortifying immune surveillance and promoting tumor control. The identification of JAML as a central mediator of XCL1 secretion by CD4+ TRMs further enriched our understanding of TRM‐driven dendritic cell mobilization, firmly positioning these cells as key orchestrators of anti‐tumor immunity.

Unlike traditional views [87, 88, 89], recent studies have revealed more complex interactions between cDC1s and CD4+ T cells [90, 91]. CD4+ T cells can “help” or “license” cDC1s to induce CD8+ CTL anti‐tumor responses [92, 93], whereas early CD4+ T cell activation depends on cDC1s [94]. Mechanistically, activated CD4+ T cells produce IFN‐β through the STING pathway, which enhances MHC class I cross‐presentation by cDC1s and synergizes with CD40L to optimize cDC1 costimulatory functions, thereby augmenting CTL‐mediated immunity [95]. These IFN‐I‐producing CD4+ T cells were also found to correlate with improved overall survival and PD‐1 blockade response, likely representing the CD4+ TRMs identified in our study. Furthermore, cDC1‐based vaccination markedly increases CD4+ TRMs tumor infiltration [64], a finding that is consistent with our hypothesis that cDC1s promote CD4+ TRMs retention through CXCL16‐CXCR6 signaling. Combined with our demonstration that CD4+ TRMs recruit cDC1s via the XCL1‐JAML axis, these findings support a bidirectional positive feedback loop: recruitment followed by licensing and immune memory consolidation, maximizing CD4+ TRM‐cDC1 synergy in the tumor microenvironment.

The dynamic interplay between immune activation and tumor‐induced immune escape is a defining feature of cancer progression [96]. The TME is characterized by complex cellular crosstalk between tumor cells and immune components, involving the activation of immune checkpoint pathways and the systematic disruption of effective anti‐tumor responses [97, 98]. Tumor cells actively induce dysfunction or elimination of effector immune cells to subvert immune surveillance [99], while the TME concurrently cultivates an immunosuppressive niche—accumulating regulatory T cells, tumor‐associated macrophages, and myeloid‐derived suppressor cells [100, 101]—that further attenuates immune activity. Beyond these, emerging evidence indicates that the TME also reprograms tissue‐resident immune cells into states of exhaustion or dysfunction that facilitate immune evasion [102, 103, 104]. In our study, we observed that within the immunosuppressive milieu, CD4+ TRMs manifested a dysfunctional phenotype, marked by attenuated tissue‐residency features, compromised cDC1 mobilization, and enhanced expression of inhibitory molecules. Mechanistically, we elucidate that PD‐1 signaling suppressed JAML expression in CD4+ TRMs through inhibition of the PI3K/Akt/mTOR signaling pathway, thereby curtailing XCL1 secretion and impeding cDC1 recruitment—a critical axis of tumor‐mediated immune escape. This insight unveiled novel therapeutic targets for restoring TRM functionality and reinvigorating anti‐tumor immunity.

Despite the clinical success of ICIs in advanced NSCLC, therapeutic resistance remains a formidable challenge [105, 106], and there is an unmet need for reliable biomarkers to predict treatment response [107, 108]. Our study demonstrated that the abundance and functional integrity of tumor‐infiltrating CD4+ TRMs were associated with early clinical stage, improved prognosis, and enhanced responsiveness to ICI therapy. Notably, combination therapy involving anti‐PD‐1 agents and JAML agonism not only restored JAML expression and cDC1 recruitment but also synergistically amplified cytotoxic CD8+ T‐cell responses, culminating in superior tumor control in preclinical models. These findings paved the way for integrating ICIs with targeted modulation of TRM‐associated pathways to restore the full immune potential of CD4+ TRMs in anti‐tumor responses.

Nevertheless, our study was not without limitations. The molecular mechanisms linking PD‐1 signaling to JAML downregulation, particularly intermediate signaling nodes beyond PI3K, require further elucidation. A more granular dissection of the heterogeneity within the CD4+ TRMs compartment is also necessary to fully delineate the functional nuances of these cells across diverse tumor contexts. Additionally, our preliminary observations indicate that the prognostic relevance of CD4+ TRMs is more pronounced in LUAD than in LUSC. The subtype‐specific mechanisms underlying this differential response remain unclear, and the generalizability of our findings across different NSCLC subtypes requires further validation. The translational challenges of JAML agonist strategies, including specificity, safety profiles, and optimal therapeutic protocols, also must be addressed in future preclinical studies.

In summary, our findings revealed that tumor‐infiltrating CD4+ TRMs play a pivotal role in modulating the anti‐tumor immune landscape of NSCLC through the orchestration of cDC1 recruitment through an XCL1‐JAML axis that is negatively regulated by PD‐1 signaling. These insights not only refined the definition and functional understanding of CD4+ TRMs in human cancer but also provided a robust framework for the development of novel immunotherapeutic strategies targeting CD4+ TRMs, as well as prognostic tools in NSCLC. Future studies aimed at further dissecting the molecular pathways governing TRM functionality and their intercellular interactions within the TME will be essential for translating these findings into clinical benefit.

## 4 Methods and Materials

### 4.1 Clinical Specimen Collection and Ethical Compliance

NSCLC tumor tissues (T), paired normal lung tissues (N), and fresh peripheral blood (PB) were collected from 180 patients with NSCLC who underwent curative surgical resection at the Department of Thoracic Surgery, Second Affiliated Hospital, Zhejiang University School of Medicine, between 2022 and 2025. Tumor samples were harvested from the geometric center of solid nodules with macroscopically distinct boundaries. A paired mirror‐image tissue block was immediately analyzed via intraoperative frozen section to verify the presence of viable malignant cells. Subsequently, the research specimens underwent strict pathological assessment by board‐certified pathologists to ensure adequate tumor cellularity (>70%, visual estimation) and to exclude areas of necrosis or fibrosis. Paired normal lung tissue specimens were harvested from macroscopically normal, non‐atelectatic regions located at least 5 cm distant from the tumor margin, with histological verification confirming the absence of tumor infiltration and significant inflammation. Autologous peripheral blood samples were collected via venipuncture before surgical intervention. None of the patients had received neoadjuvant chemotherapy, radiotherapy, or immunotherapy prior to surgery. The comprehensive clinicopathological characteristics of all the patients, including age, gender, NSCLC histological subtype (adenocarcinoma or squamous cell carcinoma), and TNM stage according to the AJCC Cancer Staging Manual, are detailed in Table S3. Sample allocation across all experimental procedures and figure panels is systematically documented in Table S4, which provides complete correspondence between each figure and the specific patient sample numbers used, along with sample sizes (n) for each analysis. Tissue microarray (TMA) slides (HLugS180Su02 containing 90 paired lung squamous cell carcinoma cases with adjacent non‐cancerous tissues, and HLugA180Su11 containing 90 paired lung adenocarcinoma cases with adjacent non‐cancerous tissues) were purchased from Shanghai Outdo Biotech Company. Pathological quality control of TMA cores was performed to exclude cores with artifacts, necrosis, or insufficient tumor content. These TMA cohorts represent geographically and temporally distinct patient populations independent from our fresh tissue cohort. Detailed patient demographics and clinicopathological parameters for the TMA cohorts are provided in Tables S5 and S6, respectively. All the specimens were anonymized in accordance with local ethical guidelines (as stipulated by the institutional review board) and the Declaration of Helsinki. Written informed consent was obtained from each participant prior to specimen collection. The study protocol was approved by the Ethics Review Board of the Second Affiliated Hospital, Zhejiang University School of Medicine (Ethics approval number: C20240609).

### 4.2 Animal Models and Husbandry

C57BL/6J mice were purchased from the Animal Center of Slaccas (Shanghai, China). Jaml‐knockout (Jaml‐KO) mice on a C57BL/6 background were obtained from GemPharmatech (Cat. No. T037999). The Jaml‐KO genotype had been rigorously validated for purity in our previous study (Hao et al., 2024), and gene disruption was further confirmed at the protein level. Both male and female mice, aged 6–10 weeks and weighing approximately 18–25 g, were used in the experiments. The mice were raised in the SPF barrier environment of the Laboratory Animal Center of the Second Affiliated Hospital of Zhejiang University School of Medicine. All procedures were approved by the Animal Ethics Committee of the Second Affiliated Hospital of Zhejiang University School of Medicine (Animal Ethics Approval Number: 20220317A) and were conducted in accordance with established animal welfare guidelines.

### 4.3 Tumor Implantation

LLC cells (the mouse lung adenocarcinoma cell line) and CMT167 cells (fluorescently labeled, syngeneic mouse lung adenocarcinoma cell line) were obtained from the National Collection of Authenticated Cell Cultures (Shanghai, China) and cultured in Dulbecco's modified Eagle's medium (DMEM; Gibco) containing 10% fetal bovine serum (FBS) and 1% penicillin/streptomycin. The cells were maintained at 37°C in a 5% CO_2_ atmosphere. For subcutaneous tumors, 1 × 10^5^ LLC cells or 1 × 10^6^ CMT167 cells in 100 µL of PBS were injected into the flanks of C57BL/6 mice. For orthotopic lung tumors, 1 × 10^6^ CMT167‐luc cells suspended in 100 µL of sterile PBS/Matrigel (Corning) (1:1) were injected into the left lung lobe of anesthetized C57BL/6 mice via a transthoracic approach using an insulin syringe. For subcutaneous tumors, tumor volume was measured with calipers every two days; mice were euthanized once the longest diameter exceeded 15 mm or upon ulceration/cachexia. For orthotopic tumors, tumor burden was evaluated via bioluminescence imaging and body weight assessment at designated time points; Bioluminescence imaging: D‐luciferin (75 mg in 5 mL of PBS; final concentration 15 mg/mL) was administered intraperitoneally (100 µL per mouse), and the mice were imaged 5–10 min post‐injection using the IVIS imaging system. Respiratory distress served as the primary humane endpoint for euthanasia.

### 4.4 Therapeutic Interventions

For in vivo antibody treatment assays, C57BL/6 mice received intraperitoneal injections on Days 7, 9, and 11 after tumor implantation. The antibodies used were isotype control antibody (Bio X Cell, 200 µg per mouse), anti‐PD‐1 (Bio X Cell, 200 µg per mouse), anti‐CXADR (BioLegend, 100 µg per mouse), and JAML agonist (BioLegend, 100 µg per mouse). For monotherapy, each mouse was injected with 200 µL of the indicated antibody solution in PBS. For combination therapy, each antibody was prepared separately in PBS to a final volume of 200 µL and administered individually. For lymphocyte egress blockade, FTY720 (0.05mg per mouse, 200 µL i.p.) was given every other day starting on Day 1. CD4+ T cell depletion was performed using anti‐CD4 antibody (Bio X Cell, 200 µg per mouse, 200 µL i.p) administered intraperitoneally on Days 2 and 3 post‐tumor implantation only. For adoptive transfer experiments, CD4+ TRMs were isolated from the lung tissues of either wild‐type C57BL/6 or Jaml‐KO mice. The transfer was performed intravenously on Days 8 and 10 post‐tumor implantation, with 1 × 10^6^ cells in 100 µL of PBS per injection followed by intraperitoneal administration of therapeutic antibodies on Days 11, 13, and 15.

### 4.5 Tissue Dissociation and Single‐Cell Suspension Preparation

Freshly excised tissues were immediately stored in sterile RPMI 1640 (Corning) supplemented with 10% FBS (Giboco) and 1% streptomycin and penicillin (Life Technologies) and processed within 2 h. The tissues were finely chopped and then digested in RPMI 1640 containing 10% FBS, type I collagenase (1 mg/mL), and type IV collagenase (1 mg/mL) for 1 h at 37°C using the gentleMACS Dissociator (Miltenyi Biotech), following the manufacturer's protocol. The resulting single‐cell suspension was sequentially filtered through sterile 70 µm cell strainers. To remove red blood cells, 10× Red Blood Cell (RBC) Lysis Buffer (BioLegend) was diluted to a 1× working concentration with deionized water and incubated on ice for 5 min. The cell suspensions were then stored in complete medium at 4°C until further use. Peripheral blood (PB) lymphocytes were isolated by centrifugation on a Ficoll gradient.

### 4.6 Flow Cytometry Analysis and Cell Sorting

Fresh tissue cells were preincubated in a blocking solution containing PBS, 2% fetal calf serum, and 0.1% (w/v) sodium azide, along with an FcgIII/IIR‐specific antibody to prevent nonspecific binding. For cell‐surface staining, single‐cell suspensions were incubated with the indicated fluorescently conjugated antibodies at 4°C for 45 min or at room temperature for 15 min, using Cell Staining Buffer (BioLegend). All antibodies were validated according to the manufacturer's instructions. Detailed information for all antibodies used in flow cytometry is provided in Table S1. For intracellular and intranuclear staining, surface staining was performed first as described, followed by fixation of the cells in 0.5 mL/tube Fixation Buffer (BD Biosciences) for 20 min in the dark at 4°C. Fixed and permeabilized cells were then resuspended in Intracellular Staining Perm Wash Buffer (BD Biosciences) and stained with a predetermined optimal concentration of fluorophore‐conjugated antibody for 20–30 min in the dark at room temperature. After being washed with PBS, the cells were filtered through a 70‐µm mesh. The stained cell suspension was stored in Cell Staining Buffer (BioLegend) at 4°C. For intravascular CD45 staining, 3 µg of Brilliant Violet 510‐conjugated anti‐mouse CD45 (clone 30‐F11; BioLegend) was diluted in 200 µL of PBS and injected into each mouse via the tail vein. The mice were euthanized 10 min later, and peripheral blood, lung, and tumor tissues were harvested and processed into single‐cell suspensions under light‐protected conditions for subsequent flow cytometric analysis. Data were acquired on an LSRFortessa (BD Biosciences), and analysis was conducted using FlowJo (FlowJo 10.8.1). For cell sorting, CD4+ TRMs (CD45+ CD3+ CD4+ CD69+ CD103+), SP cells (CD45+ CD3+ CD4+ CD69+ CD103‐), and DN cells (CD45+ CD3+ CD4+ CD69‐ CD103‐) were isolated from tumor and paired normal lung tissues using a Human CD4 T‐Cell Isolation Kit (BioLegend) according to the manufacturer's protocol, and were subsequently sorted using a BD FACS Aria. cDC1 cells (CD45+ Lin (CD3, CD19, CD20, CD56, and CD66b) – CD14‐ HLADR+ CD11c+ CD141+) were isolated from the remaining cell suspension after CD4+ T cell enrichment. Naïve CD4+ T cells (TN) (CD45RA+ CD45RO‐ CD3+ CD4+ CD62L+ CCR7+), central memory CD4+ T cells (TCM) (CD45RA‐ CD45RO+ CD3+ CD4+ CD62L+ CCR7+), and effector memory CD4+ T cells (TEM) (CD45RA‐ CD45RO+ CD3+ CD4+ CD62L‐ CCR7‐) were isolated from peripheral blood. For dead cell exclusion, the cell pellet was resuspended in Cell Staining Buffer, and 5 µL of 7‐AAD per million cells was added. The cells were incubated in the dark for 5–10 min, and dead cells were excluded by applying a gating strategy during cell sorting. Cell populations were sorted using either the SORP‐aria or ARIA‐III instrument (BD Biosciences) and analyzed with BD FACSDIVA software (BD Biosciences) and FlowJo software (FlowJo 10.8.1). For absolute cell quantification, Count Beads (BioLegend, #424902) were added to each sample at a defined volume immediately before acquisition. Absolute cell counts were calculated according to the manufacturer's protocol, based on the basis of the ratio of bead events to cell events.

### 4.7 Immunofluorescence and mIHC Staining, Imaging, and Analysis

Tumor and normal lung tissues were collected and fixed in 4% (wt./vol) paraformaldehyde (PFA) overnight before being embedded in paraffin blocks to generate formalin‐fixed paraffin‐embedded (FFPE) tissue samples. Five‐micrometer‐thick sections were then cut, dewaxed in xylene, rehydrated through graded ethanol, and rinsed with distilled water. Antigen retrieval was performed in a microwave using EDTA antigen retrieval buffer (pH 8.0). After the addition of 3% BSA was for blocking at RT for 30 min, primary antibody (listed in Table S1) staining was performed at 4°C overnight. Following washes with PBS (pH 7.4), the appropriate secondary antibodies were applied and incubated in the dark at room temperature for 1 h. DAPI was subsequently added for 10 min at room temperature to stain the nuclei. For multiplex immunohistochemistry (mIHC), sequential staining was performed using the Opal Polaris 7‐Color Manual IHC Kit (AKOYA Biosciences). After each primary antibody incubation, the slides were treated with Opal Polymer HRP followed by the corresponding Opal fluorophore (Opal 520, 540, 570, 620, 650, 690, or 780). Between each round of staining, microwave‐based stripping using AR6/AR9 buffer was performed to remove antibody complexes while preserving fluorescent signals. For isotype control validation, parallel sections were stained following identical protocols with primary antibodies replaced by species‐ and isotype‐matched control antibodies at equivalent concentrations, maintaining all secondary reagents and Opal fluorophores unchanged. Epifluorescence multispectral images of the entire sections were acquired using a Nikon Eclipse C1 system (Nikon) and scanned at high resolution with a Panoramic SCAN II system (3DHISTECH Ltd.). For mIHC samples, images were acquired using the Vectra Polaris imaging system (AKOYA Biosciences) with spectral unmixing performed using inForm software (version 2.6) to separate individual fluorophore signals and enable quantitative analysis of cell phenotypes and spatial distributions.

Whole‐section image analysis and cell quantification were performed using QuPath software (version 0.4.3). Cell segmentation was achieved through automated nuclear detection based on DAPI staining, with cell boundaries delineated by expanding 2–5 µm from nuclear centroids according to local cytoplasmic marker intensity profiles. Cell phenotyping was accomplished using multi‐channel fluorescence intensity thresholding algorithms, where positive marker expression was defined as signal intensity exceeding background fluorescence by a minimum threshold determined individually for each marker channel through histogram analysis and validated against manual annotations. Multi‐positive cell populations were identified through Boolean logic operations combining individual marker positivity criteria. Cell densities were calculated as total cell counts per mm^2^ of analyzed tissue area. Spatial analysis was performed by calculating nearest neighbor distances between distinct cell populations using centroid‐to‐centroid Euclidean distance measurements. For mIHC samples processed with inForm software (version 2.6), spectral unmixing was first performed to generate single‐channel images for each fluorophore, followed by adaptive nuclear segmentation algorithms and machine learning‐based phenotyping classifiers trained on manually annotated representative regions to identify complex co‐expression patterns and quantify spatial relationships between distinct cell populations across the entire tissue section.

Antibodies for immunofluorescence staining were as follows: **Human**: anti‐CD4 (Abcam, ab133616, clone EPR6855, 1:500), anti‐CD103 (Abcam, ab224202, clone EPR22590‐27, 1:200), anti‐CD69 (Abcam, ab233396, clone EPR21814, 1:100), anti‐XCL1/Lymphotactin (LifeSpan BioSciences, LS‐B5938, clone 1E1, 1:200), anti‐XCL1/XCL2 (Abcam, clone EPR26181‐30, 1:100), anti‐HLA‐DR (Abcam, ab92511, clone EPR3692, 1:100), anti‐XCR1 (Antibodies‐online, ABIN6266058, clone not specified, 1:100), anti‐pan‐Cytokeratin (Abcam, ab7753, clone C‐11, 1:200), anti‐CD68 (Abcam, ab213363, clone EPR20545, 1:200), and anti‐CD11c, C‐terminal (Abcam, ab52632, clone EP1347Y, 1:200); **Mouse**: anti‐CD4 (Abcam, ab183685, clone EPR19514, 1:300), anti‐CD8α (Abcam, ab217344, clone EPR21769, 1:300), anti‐CD103 (Abcam, ab224202, clone EPR22590‐27, 1:1000), anti‐Granzyme B (Abcam, ab255598, clone EPR22645‐206, 1:3000), anti‐Perforin (Abcam, ab16074, clone CB5.4, 1:600), anti‐XCL1/Lymphotactin (R&D Systems, AF486, polyclonal, 1:200), anti‐XCR1 (BioLegend, clone ZET, cat. 148202, 1:100), and anti‐CLEC9A (Abcam, ab300433, clone EPR24271‐117, 1:100); **Isotype controls** (Abcam) included mouse IgG1 (ab91353, clone B11/6), mouse IgG2b (ab170192, clone 7E10G10), mouse IgG2a (ab18415, clone MG2a‐53), rabbit IgG (ab172730, clone EPR25A), rat IgG2a (ab18450, clone RTK2758), and goat IgG (ab37373, polyclonal); isotypes were used at Ig concentrations matched to their corresponding primaries.

### 4.8 H&E Staining and Image Acquisition

Tumor tissues and adjacent normal lung tissues were fixed in 10% formalin overnight, dehydrated through graded alcohols, cleared in xylene, and embedded in paraffin blocks. Five‐micrometer‐thick sections were cut and mounted on glass slides and then incubated at 60°C for 2 h. Slides were deparaffinized in xylene (3 × 5 min), rehydrated through a descending ethanol series (100%, 95%, 70%; 3 min each), and rinsed in distilled water. Sections were stained with hematoxylin for 5 min, differentiated in acid alcohol, blued in tap water, counterstained with eosin for 2 min, dehydrated, cleared, and mounted. Digital whole‐slide images were acquired at 40× magnification using the PANNORAMIC 250 Flash III digital slide scanner (3DHISTECH) and analyzed using CaseViewer software (3DHISTECH).

### 4.9 Quantitative Real‐Time PCR (qRT‐PCR)

Tumor tissue was lysed by the addition of 1 mL of TRIzol Reagent. RNA was then extracted from the aqueous phase using phenol‐chloroform extraction, and the RNA precipitate was subsequently washed with 1.5 mL of 75% ethanol. Next, 4 µL of 5× EasyScript All‐in‐One SuperMix for qPCR and 1 µL of gDNA Remover were added to synthesize cDNA via reverse transcription. Finally, mRNA transcripts were quantified via quantitative PCR using SYBR Green Master Mix (Bio‐Rad Laboratories) and normalized to β‐actin. The sequences of the primers used were as follows: Actb: forward, 5′‐GATCTGGCACCACACCTTCT‐3′; reverse, 5′‐GGGGTGTTGAAGGTCTCAAA‐3′; *CXADR*: forward, 5′‐AATGGCTGATATCCCCGTCT‐3′; reverse, 5′‐ATAGATGCGTCGCCAGACTT‐3′; *JAML*: forward, 5′‐AGAGCACGCCAAGGACGAATA‐3′; reverse, 5′‐GGAGCAGGAGAGAGCCATCAT‐3′; *PDCD1*: forward, 5'‐ AAGGCGCAGATCAAAGAGAGCC‐3'; reverse, 5'‐ CAACCACCAGGGTTTGGAACTG‐3'.

### 4.10 Enzyme‐Linked Immunosorbent Assay (ELISA)

To confirm the secretion of XCL1 by CD4+ TRMs, single‐cell suspensions of FACS‐sorted CD4+ TRMs (DP), SP, and DN cells were prepared using flow cytometry for cell sorting, as described previously. The suspensions were then divided into aliquots, each containing 100,000 cells. XCL1 concentrations were measured using an ELISA kit (CUSABIO, #CSB‐E08712h), following the manufacturer's protocol. The assay was performed in conjunction with the Limulus Amebocyte Lysate (LAL) assay QCL‐1000 (Lonza, Valais, Switzerland).

### 4.11 In Vitro Cell Culture and Intervention Assays

For the costimulation assay, CD4+ TRMs were plated in 96‐well round‐bottom tissue culture plates at 1 × 10^5^ cells/mL (2 × 10^4^ cells/200 µL/well) and stimulated with anti‐CD3 (BioLegend) at a concentration of 10 µg/ml. The cells were divided into a blank group or treated with 10 µg/ml of anti‐CD28 (BioLegend) or 10 µg/ml of recombinant human CXADR chimera. The cells were incubated at 37 °C for 48 h, followed by two washes with cell staining buffer (BD eBiosciences) before flow cytometry (FACS) analysis. To measure the production of the intracellular chemokine XCL1, 4 µg/ml of brefeldin A (BFA) (Multi Science) was added to each well for the final 5 h of incubation, followed by staining and flow cytometric analysis. For the PD‐1 pathway inhibition assays, FACS‐sorted CD4+ TRMs were plated in 96‐well round‐bottom tissue culture plates at 1 × 10^5^ cells/mL (2 × 10^4^ cells/200 µL/well) and co‐cultured with anti‐CD3 and anti‐CD28 at a concentration of 10 µg/ml, with or without 1 µg/ml of recombinant human PD‐L1 (BioLegend), at 37°C for 48 h, followed by downstream assays. For the whole‐cell suspension anti‐PD‐1 blocking assays, fresh tissue was dissociated into single‐cell suspensions using collagenase. The cells were plated in 24‐well round‐bottom tissue culture plates (1 × 10^6^ cells/500 µl/well) with sterile RPMI 1640 (Corning), 10% FBS (Gibco), and 1% streptomycin and penicillin (Life Technologies). Cultures were treated with human anti‐PD‐1 antibody (5 µg/mL; BioLegend) or the corresponding isotype control for 48 h at 37°C in 5% CO_2_, after which downstream assays were performed. For the recruitment assays, CD4+ TRMs were subsequently sorted from the cultured cells and used in the cDC1 recruitment experiments. For the PI3K‐pathway validation assays, FACS‐sorted CD4+ TRMs were plated in 96‐well round‐bottom tissue culture plates at 1 × 10^5^ cells/mL (2 × 10^4^ cells/200 µL/well) and co‐cultured with anti‐CD3 and anti‐CD28 at a concentration of 10 µg/ml, with either LY294002 (5 µg/mL, Beyotime) or SC79 (10 µg/mL, Beyotime) at 37°C for 48 h.

### 4.12 Cell Chemotaxis and Recruitment Assay

Single‐cell suspensions obtained by FACS sorting of CD4+ TRMs (DP), SP, and DN populations were maintained in complete medium (RPMI‐1640 supplemented with 10% FBS and 1% penicillin‐streptomycin). Chemotaxis was assessed using 5 µm pore transwell inserts (Corning, 3421). Sorted CD4+ T cell subsets (1 × 10^5^ cells/400 µL) were placed in the lower chamber and stimulated with PMA/ionomycin (Multi Science; 1 µL/200 µL). FACS‐sorted cDC1s (2 × 10^4^ cells/200 µL) were added to the upper chamber in suspension. After incubation at 37°C for 24–48 h, migrated cDC1s in the lower chamber were quantified by flow cytometry. For blocking experiments, anti‐XCL1 neutralizing antibody (R&D Systems; 3 µg/mL) was added to the lower chamber, with an isotype antibody control processed in parallel. All the other steps were performed as described above.

### 4.13 siRNA‐Mediated Gene Knockdown

For target gene silencing, three pairs of chemically synthesized siRNA sequences were designed and purchased from Tsingke Biotech (Beijing, China). Negative control siRNA (scrambled sequence with no homology to the mouse genome) was included as a control. siRNAs were reconstituted in RNase‐free water to a stock concentration of 20 µm and stored at ‐20°C in aliquots.

Primary CD4+ T cells were transfected using Lipofectamine 2000 (Invitrogen) according to the manufacturer's protocol. Briefly, cells were seeded at 0.5–2 × 10^5^ cells per well in 24‐well plates in antibiotic‐free medium 24 h before transfection. siRNA (50 nM final concentration) and transfection reagent (1 µL per well) were separately diluted in 50 µL Opti‐MEM, combined after 5 min, and incubated for 20 min at room temperature. The transfection complexes were added to the cells and incubated for 48–72 h. Target gene expression was assessed by flow cytometry, and the knockdown efficiency was 60–70% greater than that of the scrambled control. These knockdown cells were subsequently used for functional assays.

### 4.14 CyTOF Data Debarcoding and Analysis

Mass cytometry data, previously generated and reported in our earlier study, were reanalyzed for the present work. Raw CyTOF files were first debarcoded using a doublet filtering scheme with mass‐tagged barcodes and then manually gated to retain live, singlet, and valid immune cells. Data generated from different batches were normalized using the bead normalization method. To obtain accurate immune subset information, we applied the phenograph algorithm to all the samples. All cell events from each individual sample were pooled and included in the analysis.

### 4.15 Bulk RNA‐seq Pipeline and Bioinformatics Analysis

Total RNA was extracted from isolated lymphocytes using TRIzol reagent and isolated using TRIzol‐chloroform extraction. The aqueous phase containing RNA was collected, purified with the RNeasy MinElute Cleanup Kit (QIAGEN) according to the manufacturer's instructions, and submitted to the Institute for Library Preparation (Clontech ZapR) and Sequencing (Illumina NextSeq500). Sequencing data were filtered using SOAPnuke by: (1) removing reads containing sequencing adapters; (2) removing reads with a low‐quality base ratio (base quality ≤ 5) greater than 20%; and (3) removing reads with an unknown base (‘N’ base) ratio exceeding 5%. Clean reads were subsequently obtained and stored in FASTQ format. These clean reads were mapped to the reference genome using HISAT2. All downstream analyses were conducted in R (v4.2.1) with Bioconductor 3.15. Differential expression analysis was performed using limma (v3.52.2) and edgeR (v3.38.4). Genes with a log_2_(fold change) ≥ 1, expression in >10% of cells, and P < 0.05 were considered differentially expressed. Functional enrichment analysis was performed for Gene Ontology (GO), Kyoto Encyclopedia of Genes and Genomes (KEGG), and Hallmark gene sets using the enrichplot package (v1.16.1), applying a hypergeometric test for statistical evaluation. Data visualization included volcano plots generated with EnhancedVolcano (v1.14.0), heatmaps generated with pheatmap (v1.0.12), and principal component analysis (PCA) performed with plotly (v4.10.0) to generate both 2D and 3D PCA plots for sample clustering.

### 4.16 Single‐Cell RNA‐Seq Data Analysis

We obtained the metadata for quality‐controlled and normalized single‐cell RNA‐seq data from the GEO public databases, including GSE127465, GSE131907, GSE136246, GSE148071, and GSE210347. The R package Harmony (version 1.2.3) was used to integrate the data from different sample batches, followed by dimensionality reduction and clustering with Seurat (version 4.4.1). We employed the shared nearest neighbor (SNN) graph‐based clustering algorithm (using the FindClusters function with resolution = 0.8) to delineate distinct cell clusters. These clusters were subsequently annotated on the basis of the expression of canonical lineage markers for major immune and non‐immune cell populations, and their distributions were visualized using UMAP plots. Specific T‐cell subsets were further refined using combinations of phenotypic and functional markers, with module scoring performed using the AddModuleScore function in Seurat. Further reclustering analyses were performed using corresponding marker genes to refine cell identities. For stratification analysis, cells within specific subpopulations were classified into high‐ and low‐ expression groups on the basis of the median expression cutoff of target genes. Cell‒cell correlation analysis was conducted using Spearman correlation coefficients to assess co‐occurrence patterns between cell populations across samples. Cell‒cell communication analysis was performed using CellChat (v1.6.1) to systematically identify and quantify ligand‒receptor interactions between cell types. Differential interaction strength was calculated by comparing tumor versus normal tissue contexts. Transcription factor activity inference was performed using SCENIC (v1.3.0) with the RcisTarget and AUCell packages to identify active regulatory networks and score regulon activity across cell populations. Pathway activity analysis was conducted using single‐sample gene set enrichment analysis (ssGSEA) implemented in the GSVA package (v1.44.2). Differential gene expression analysis between cell subpopulations was performed using the FindMarkers function with the Wilcoxon rank sum test. Visualization methods included heatmaps (ComplexHeatmap v2.12.0), volcano plots (EnhancedVolcano v1.14.0), dot plots, and feature plots for marker expression patterns.

### 4.17 Spatial Transcriptomics Analysis

Spatial transcriptomics data were obtained from ArrayExpress (accession E‐MTAB‐13530), comprising 10X Visium spatial gene expression profiles from 8 paired NSCLC tumor and adjacent normal lung tissues (20 tumor sections and 16 normal sections). Raw spatial data were processed using Space Ranger (10X Genomics) for alignment and spot detection. Cell type deconvolution was performed using Cell2Location (v0.1.3) with the integrated single‐cell RNA‐seq reference atlas described above as input. The deconvolution analysis estimated cell type abundances for each spatial spot, generating a cell type × spot matrix. SpaCET (v1.0.0) was utilized for downstream spatial analysis. Cell‒cell colocalization was assessed using Spearman correlation coefficients between cell type abundances across spatial spots, with statistical significance determined by permutation testing (*n* = 1000). Spatial proximity analysis was performed to quantify spatial relationships between cell types. First, enriched spots for each cell type were identified on the basis of the Cell2Location deconvolution results (abundance threshold > 0.05). For source‐target cell type pairs, Euclidean distances from all source‐enriched spots to their nearest target‐enriched spot were calculated and averaged to determine the recruitment distance. Distances were computed in Visium platform spatial coordinates and subsequently converted to micrometers (interspot center distance of approximately 100 µm). To assess recruitment specificity, permutation testing (*n* = 1000 iterations) was performed by randomly shuffling target cell type spatial positions, recalculating distances, and generating a null distribution. The observed mean distances were compared against permutation distributions to calculate empirical *p*‐values. *p*‐values < 0.05 with observed distances below the permutation distribution median indicated significant spatial recruitment relationships. For multicell type comparative analysis, the recruitment strength of multiple source cell types toward the same target was systematically evaluated. Distance distribution curves were generated using kernel density estimation, with bar plots displaying the mean recruitment distances and statistical significance for different source cells. A cell‒cell distance matrix was constructed where each element represented the mean spatial distance (in micrometers) between corresponding cell pairs. Shorter distances indicated stronger spatial proximity, suggesting potential recruitment or interaction relationships. Additionally, visualization was performed using the ggplot2 and ComplexHeatmap packages.

### 4.18 Tissue Microarray Analysis and Prognostic Evaluation

Two commercially available tissue microarray (TMA) slides (No. HLugS180Su02 and No. HLugA180Su11, Outdo Biotech) were purchased for immunofluorescence analysis. Each TMA slide contained 180 tissue cores (1.5 mm diameter, approximately 1.77 mm^2^ area per core), comprising 90 paired cases of lung squamous cell carcinoma (HLugS180Su02) or lung adenocarcinoma (HLugA180Su11) with matched adjacent non‐cancerous tissues. The tissue microarrays were stained using the standard multi‐color immunofluorescence protocol with a three‐marker, four‐color panel (CD4, CD103, XCL1, and DAPI). Slide images were captured with 3D Histech and analyzed using Media Cybernetics and Visiopharm software.By integrating detailed survival information, we assessed the correlation of CD4+ T cells abundance, CD4+ TRMs abundance, and XCL1 expression on CD4+ TRMs with survival prognosis in patients with NSCLC. Survival analysis was performed using the survival (v3.5.5) and survminer (v0.5.0) packages in R. Optimal cutoff values for CD4+ T cells, CD4+ TRMs, and XCL1+ CD4+ TRMs were determined using the surv_cutpoint function from the survminer package, which employs the maximally selected rank statistics method (Lausen and Schumacher). This approach systematically evaluates all possible cutoff points and selects the threshold yielding the most significant survival difference based on log‐rank statistics. Only cutoffs with statistical significance (*p* < 0.05) were considered valid. Patients were stratified into high‐ and low‐ expression groups according to these cutoff values. Kaplan‒Meier survival curves were generated, and log‐rank tests were performed to evaluate differences in overall survival (OS) between groups. Multivariate Cox proportional hazards regression analysis was conducted using the ezcox package (v1.0.2) to assess the independent prognostic value of each marker while adjusting for clinical covariates, including age (<50 vs. ≥50 years), gender, tumor grade (Grades 1–3), and TNM stage (I–IV). Hazard ratios (HRs) with 95% confidence intervals (CIs) were calculated, with the low‐expression group serving as a reference. Forest plots were generated using the forestploter package to visualize multivariate Cox regression results. Statistical significance was set at *P* < 0.05.

### 4.19 Online Database Analysis

To analyze overall survival (OS) data for patients with lung adenocarcinoma (LUAD) and lung squamous cell carcinoma (LUSC), we utilized the public database TCGA (The Cancer Genome Atlas). Survival plots were generated using the online platforms Gene Expression Profiling Interactive Analysis (GEPIA) (http://gepia.cancer‐pku.cn/) and Kaplan‒Meier Plotter (www.kmplot.com). Patient samples were stratified into two groups based on the basis of the optimal cutoff or median value and evaluated using GEPIA or Kaplan‒Meier survival charts. Hazard ratios (HRs) with 95% confidence intervals (CIs) and log‐rank p‐values were computed. Spearman's correlation analysis between individual or multiple genes was performed on the basis of their average expression values. The human JAML protein interaction network and functional figure were generated using the GeneMANIA database (http://genemania.org). The correlation between JAML expression and immune infiltration in NSCLC (LUSC and LUAD) was analyzed using TISIDB (http://cis.hku.hk/TISIDB/index.php).

### 4.20 Statistical Analysis

Statistical analysis was conducted using Prism version 9.0 (GraphPad Software). Unpaired or paired Student's t‐tests were used for comparisons between two groups. One‐way ANOVA with Tukey's post hoc test was applied for multiple group comparisons. Spearman correlation was employed for correlation analyses. The data were presented as the mean ± SD, and P values less than 0.05 were considered to indicate statistical significance (*p < 0.05, **p < 0.01, ***p < 0.001, and ****p < 0.0001).

## Author Contributions

**Zheyu Shao**: performed the experiments. **Zheyu Shao** and **Zhongwei Xin** collected the samples and the clinical data. **Zheyu Shao**, **Qinyuan Liu**, **Zhiyao Zhou**, **Mingjie Lin**, **Di Chen**, **Zhixing Hao**, **Yongyuan Chen**, **Shuyang Zhang**, **Xiaoke Chen**, **Wei Lin**, and **Yuhao Lu** analyzed the data. **Xia Xu** and **Jinfan Li** performed pathological evaluation of tissue specimens. **Zheyu Shao**, **Pin Wu**, and **Dang Wu** designed the study, interpreted the results, and drafted the manuscript. **Pin Wu** and **Dang Wu** supervised the project and cowrote the paper. **Pin Wu** secured the funding. All the authors reviewed and edited the manuscript.

## Conflicts of Interest

The authors declare no conflict of interest.

## Supporting information




**Supporting File 1**: advs73591‐sup‐0001‐SuppMat.pdf.

**Supporting File 2**: advs73591‐sup‐0002‐Tables.xlsx.

## Data Availability

The Bulk RNA‐seq data reported in this study have been deposited in the Genome Sequence Archive in the National Genomics Data Center (https://ngdc.cncb.ac.cn/omix) under the accession number OMIX004492. The raw mass spectrometry flow cytometry data reported in this study have been deposited in the NGDC under the accession number OMIX003278. The single‐cell RNA‐seq datasets used were obtained from the Gene Expression Omnibus (GEO, https://www.ncbi.nlm.nih.gov/geo/), including GSE127465, GSE131907, GSE136246, GSE148071, and GSE210347. Spatial transcriptomics data were obtained from ArrayExpress (https://www.ebi.ac.uk/arrayexpress/) under accession number E‐MTAB‐13530. All the code scripts and additional data are available upon request from the corresponding author.
