# Supplementary material for: PD‐1 Inhibits CD4+ TRM‐Mediated cDC1 Mobilization via Suppressing JAML in Human NSCLC
Source: Adv Sci (Weinh). 2026 Jan 4;13(35):e07647. doi: 10.1002/advs.202507647 (PMC13292177; doi:10.1002/advs.202507647)
Supplement: Supplementary file 1 — Supporting File 1: advs73591‐sup‐0001‐SuppMat.pdf. [file ADVS-13-e07647-s001.pdf]

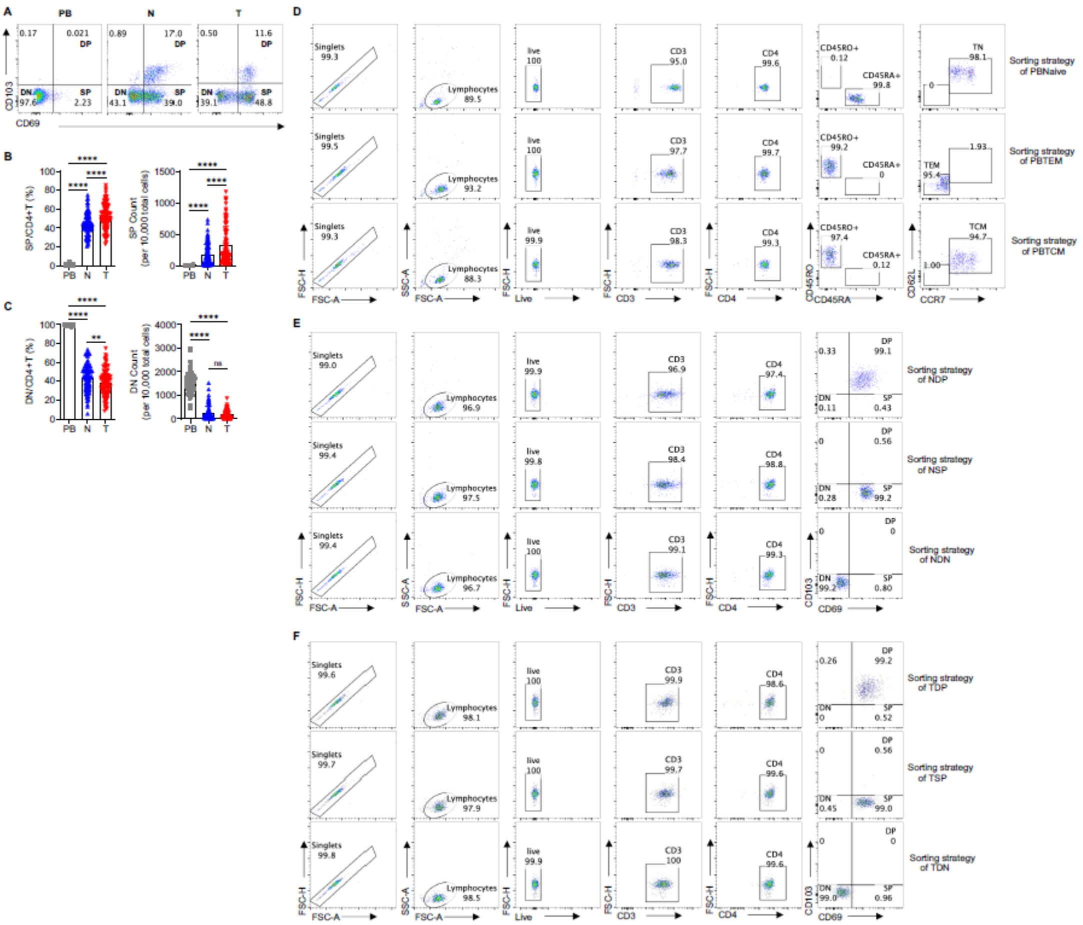

Extended Data Fig. 1 Gating and sorting of CD4+ T cell subsets in peripheral blood, normal tissue, and tumor lung tissue

A, Representative flow-cytometry plots gating CD69 and CD103 on CD4 T cells to define TRM states: DP (CD69+CD103+), SP (CD69+CD103−), and DN (CD69−CD103−), shown for PB, N, and T. **B-C,** Frequencies (left) and absolute counts (right) of SP (**B**) and DN (**C**) among CD4 T cells in peripheral blood (PB), normal lung (N), and tumor (T) from patients with NSCLC; counts were normalized per 10,000 total cells. **D-F**, Sorting strategies after sequential gating on singlets, lymphocytes, live cells, CD3, and CD4. **D**, PB subsets (TN, TCM, and TEM) defined by CD45RA and CCR7 expression. **E**, Normal-lung TRM subsets (NDP, NSP, and NDN) defined by CD69 and CD103 expression. **F**, Tumor TRM subsets (TDP, TSP, and TDN) defined by CD69 and CD103 expression. The data in (B, C) are expressed as mean ±SD. Statistical

significance was determined using one-way ANOVA with Tukey's post hoc test (B, C). ns, non-significant; *p < 0.05; ** p < 0.01; *** p < 0.001; **** p < 0.0001.

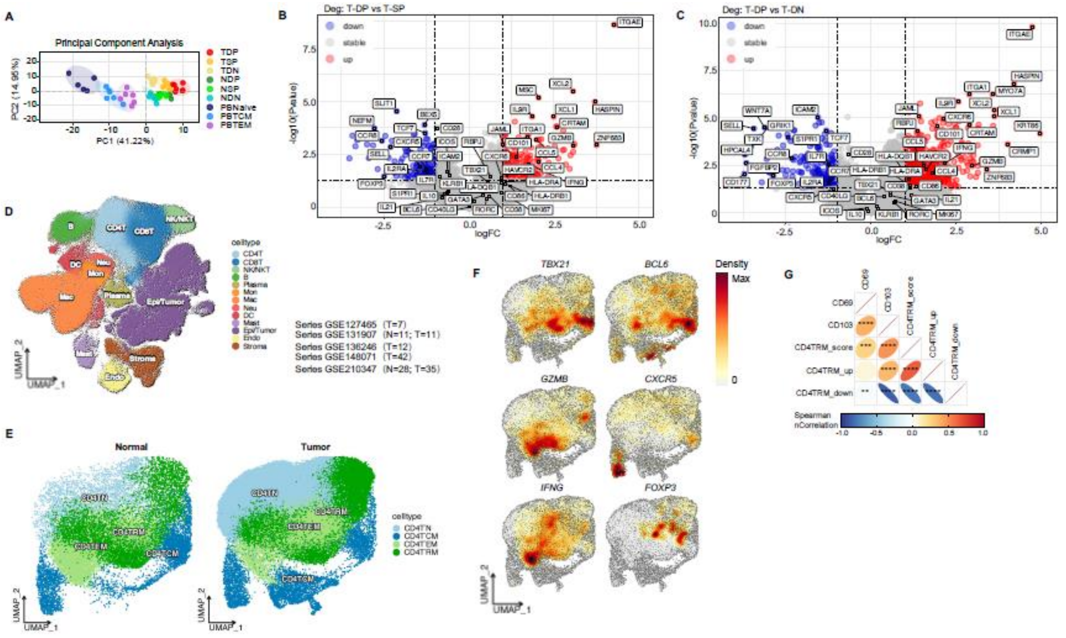

Extended Data Fig. 2 Transcriptional profiling of CD4+ T cells and CD4+ TRMs in NSCLC

A, Two-dimensional principal component analysis (2D PCA) of Bulk RNA-seq data from nine subpopulations of CD4+ T cells in peripheral blood (PBNaive, PBTCM, PBTEM), normal tissue (NDP, NSP, NDN), and tumor tissue (TDP, TSP, TDN). N, normal tissue; T, tumor; PB, peripheral blood. **B-C,** Volcano plots showing differentially expressed genes (DEGs) between TDP and TSP (B) or TDP and TDN (C). DEGs were defined as those for which P < 0.05 and |logFC| > 1. **D,** UMAP of integrated scRNA-seq data from NSCLC tumors and matched normal lung tissues, showing epithelial, endothelial, stromal, and immune cell lineages. **E,** UMAP visualization of CD4+ T-cell subtypes in paired normal and tumor tissues, highlighting the TRM, TCM, TEM, and TN clusters.

F, Feature plots showing the distribution and expression density of *TBX21, BCL6, GZMB, CXCR5, IFNG, and FOXP3*. **G,** Correlation heatmap of TRM markers and module scores. The variables included CD69, CD103, CD4TRM_up, CD4TRM_down, and the composite CD4TRM_score (up-signature score minus down-signature score). The up signature consists of *ZNF683, CXCR6, ITGA1,* and *RUNX3*, while the down signature consists of *SELL, CCR7, S1PR1,* and *ICAM2*. The color denotes the Spearman correlation coefficient, and the asterisks indicate significance.

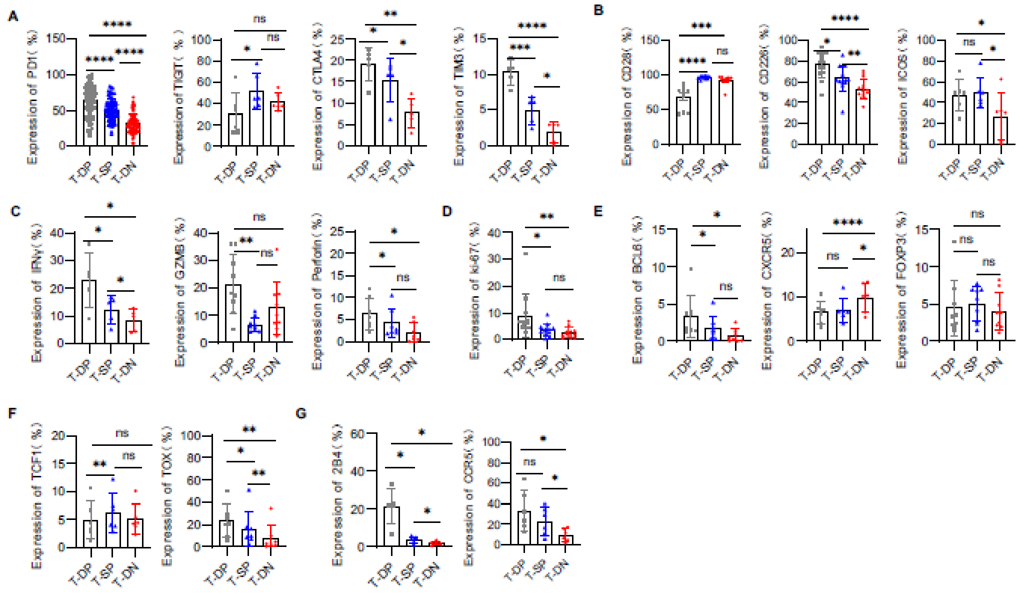

Extended Data Fig. 3 Phenotypic characteristics of tumor-infiltrating CD4+ TRMs

A-G, FCM analysis evaluating markers of inhibition (A), costimulation (B), TH1 cytokines/cytotoxicity (C), proliferation (D), TFH (E), Treg (E), differentiation (F) and residency (G) in the TDP, TSP, and TDN subsets. The data in (A-G) are expressed as the mean ± SD. Statistical significance was determined using one-way ANOVA with Tukey's post hoc test (A-G). PD-1 (n=94), TIGIT (n=6), CTLA-4 (n=5), TIM-3 (n=5), CD28 (n=10), CD226 (n=14), ICOS (n=6), IFNγ

(n=5), GZMB (n=9), Perforin (n=7), Ki-67 (n=12), BCL6 (n=8), CXCR5 (n=6), FOXP3 (n=9), TCF1 (n=6), TOX (n=7), 2B4 (n=5), and CCR5 (n=6). ns, non-significant; *$p < 0.05$; ** $p < 0.01$; *** $p < 0.001$; **** $p < 0.0001$.

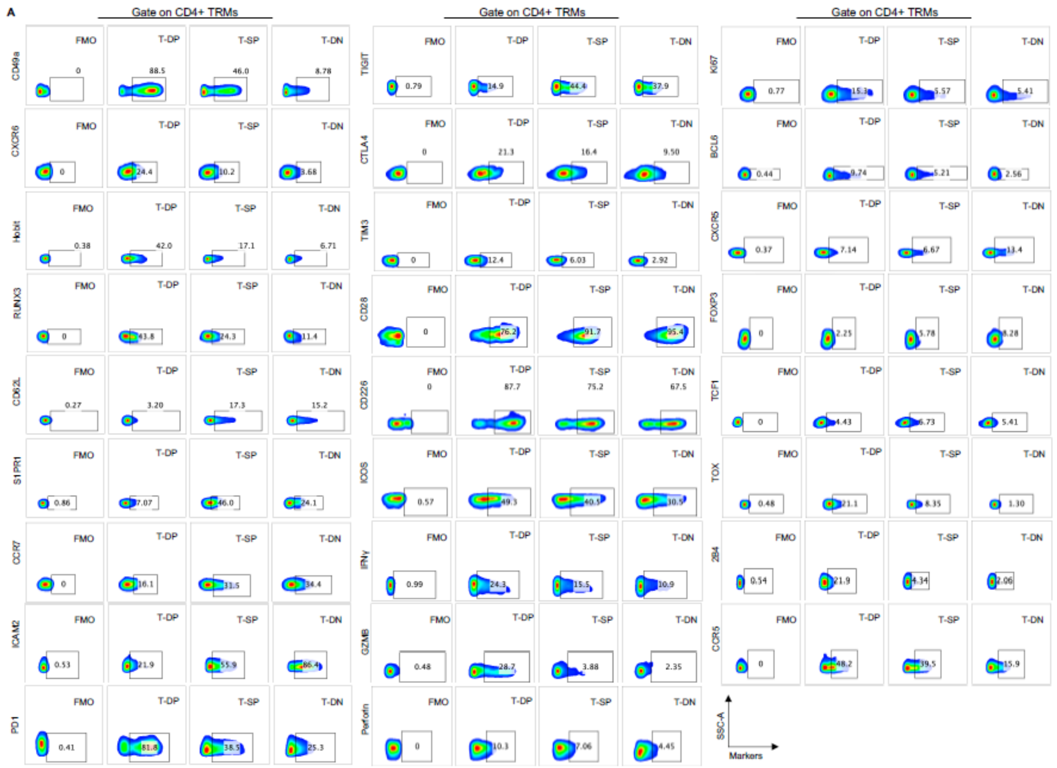

Extended Data Fig. 4 Representative FCM plots of the phenotypic characteristics of CD4+ TRMs

A, Representative FCM plots displaying the expression profiles of markers associated with inhibition, costimulation, proliferation, cytotoxicity, TH1 cytokines, T follicular helper (TFH) function, regulatory T-cell (Treg) identity and tissue-residency across the TDP, TSP, and TDN subsets. Plots were gated on CD4+ T cells. The numbers indicate the corresponding percentages.

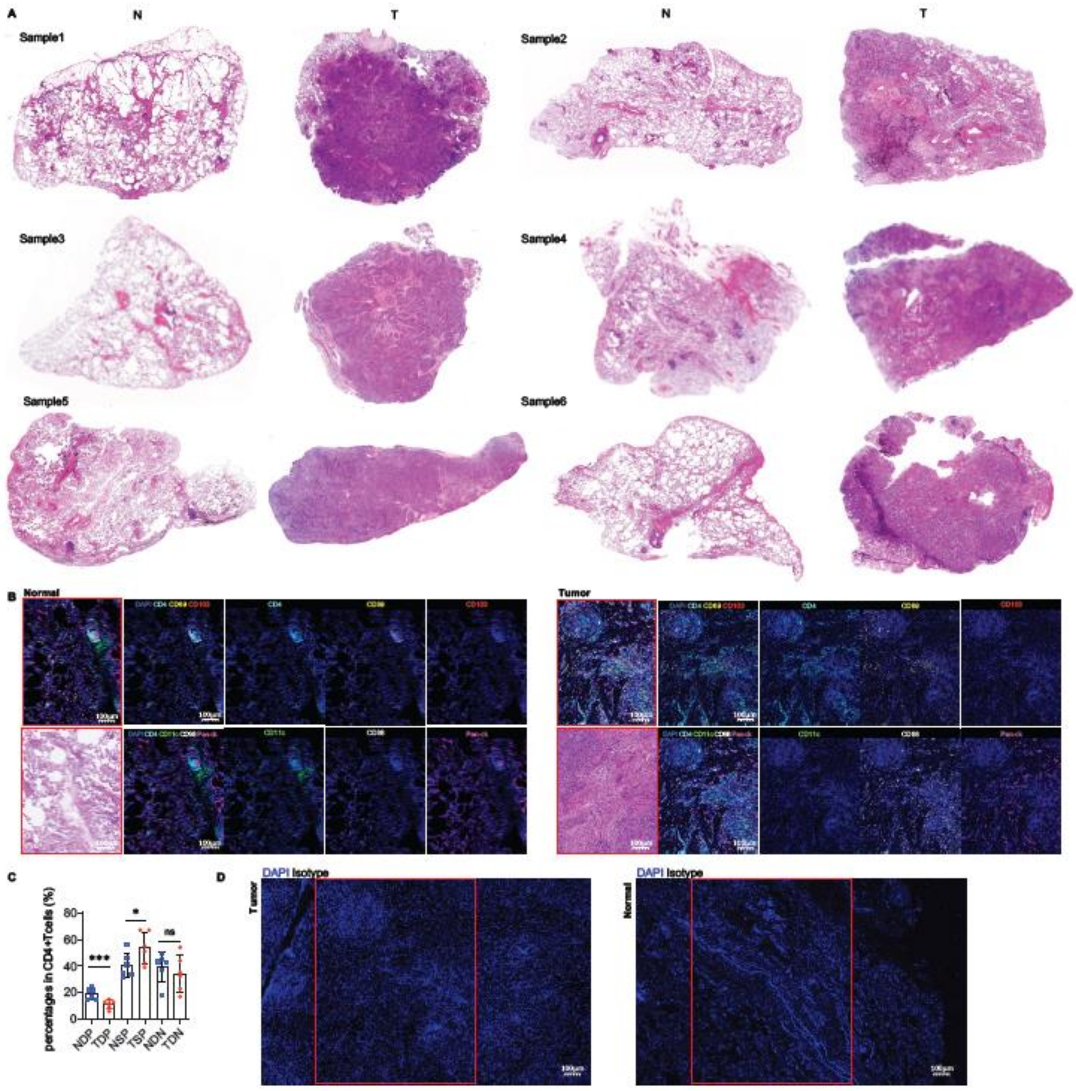

Extended Data Fig. 5 Histopathology and multiplex immunofluorescence validation of paired normal and tumor lung tissues

A, Representative whole-slide H&E-stained sections from matched normal (N) and tumor (T) lungs of from patients with NSCLC (Samples 1-6, n=6). **B,** Representative multiplex immunofluorescence images showing individual marker channels in normal lung tissue (left panels) and NSCLC tumor tissue (right panels) from Sample4. Staining shows CD4 (cyan), CD69 (yellow), CD103 (red), CD11c (green), CD68 (white), Pan-CK (magenta), nuclei (DAPI, blue), and merged images. H&E-stained sections are shown in the first image of each row. Scale bars, 100 µm. Corresponds to staining in Fig. 1K. **C,** Quantification of CD4+ T cell subset frequencies in normal (N, blue) and tumor

(T, red) tissues by whole-slide multiplex immunofluorescence analysis (n=6). DP (CD4+ CD69+ CD103+), SP (CD4+ CD69+ CD103−), DN (CD4+ CD69− CD103−). **D,** Negative control multiplex immunofluorescence showing nuclei (DAPI) with isotype control only, in tumor (left) and normal (right) tissues (Sample 4). Scale bars, 100 μm. Corresponds to the staining in Fig. 1K. The Data in (C) are expressed as the mean ±SD. Statistical analyses were performed using paired Student's t-tests (C). ns, non-significant; *p < 0.05; **p < 0.01; ***p < 0.001; ****p < 0.0001.

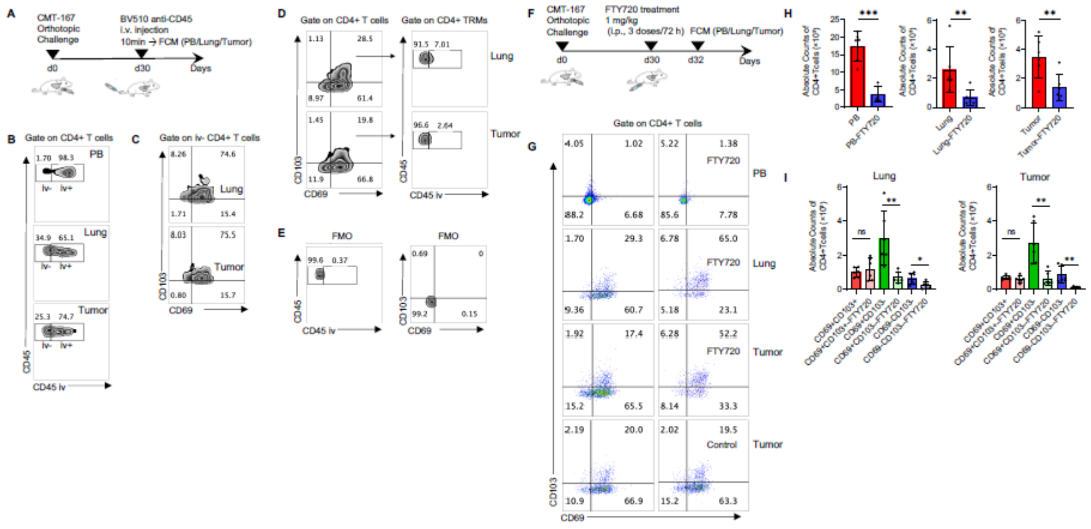

Extended Data Fig. 6 In vivo validation of CD4+ TRMs residency

A, Experimental timeline: C57BL/6 mice were orthotopically implanted with CMT-167 cells and intravenously injected with BV510-anti-CD45 antibodies on Day 30, and then peripheral blood (PB), lung tissue, and tumor tissue were analyzed by flow cytometry 10 min later. **B,** FCM gating of CD4+ T cells showing intravascular antibody labeling (CD45 iv+) in peripheral blood (PB), lung, and tumor tissues. **C,** FCM gating of iv− CD4+ T cells showing CD69 and CD103 expression in the lung and tumor. **D,** FCM analysis of CD4+ T cells. Left, Identification of CD69+CD103+ CD4+ T cells in the lung and tumor. Right, Intravenous CD45 labeling (CD45 iv+ vs. iv−) of CD69+CD103+ CD4+ T cells

to assess circulation exclusion. **E,** Fluorescence minus one (FMO) controls used to define gating boundaries for intravenous CD45 labeling (left) and CD69/CD103 expression (right). **F,** Experimental timeline: C57BL/6 mice were orthotopically implanted with CMT-167 cells and treated intraperitoneally with FTY720 (1 mg/kg) on Days 30, 31, and 32. Flow cytometry of PB, lung, and tumor tissues was performed on Day 32. **G,** Representative FCM plots gated on CD4+ T cells from PB, lung, and tumor tissues from FTY720-treated and control mice, showing CD69 and CD103 expression. **H,** Quantification of absolute CD4+ T-cell counts in PB, lung, and tumor tissues from control and FTY720-treated mice. **I,** Quantification of absolute counts of CD4+ T-cell subsets (CD69+CD103+, CD69+CD103−, and CD69−CD103−) in lung and tumor from control and FTY720-treated mice. The data in (H, I) are expressed as the mean ± SD. Statistical analyses were performed using unpaired Student's t-tests (H, I). ns, non-significant; *$p < 0.05$; **$p < 0.01$; ***$p < 0.001$; ****$p < 0.0001$.

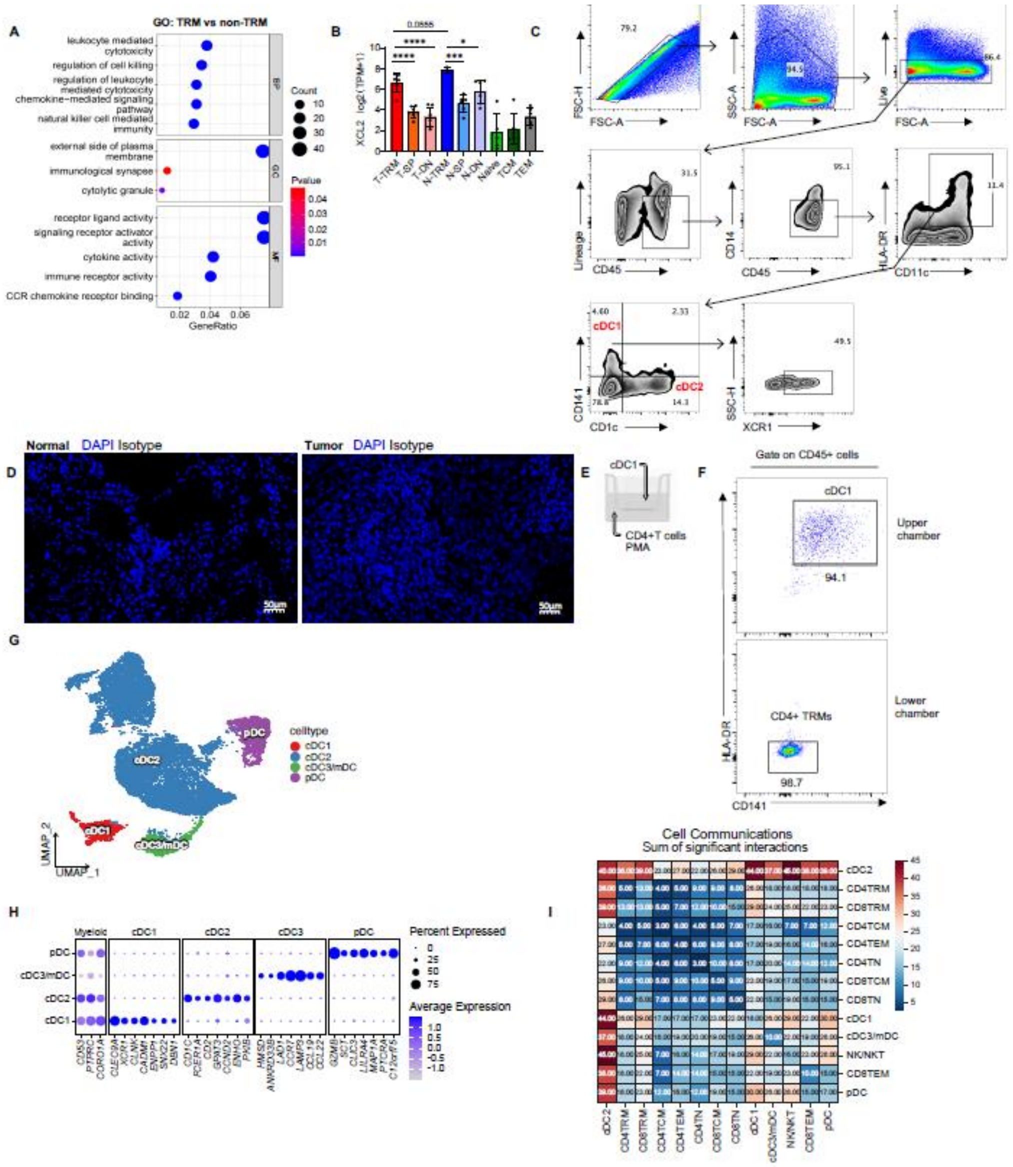

Extended Data Fig. 7 Tumor-infiltrating CD4+ TRMs display cDC1 mobilization

A,Gene Ontology (GO) enrichment analysis comparing tumor-resident CD4+ TRMs (TDP) with non-TRM (TSP/TDN). Bubble plots showing the top five of biological process (GO-BP), cellular component (GO-CC), and molecular function (GO-MF) categories. P value<0.05. **B,** Bar plots revealing the log_2(TPM+1) values of XCL2 among CD4+ T cells subpopulations based on Bulk RNA-seq. **C,** Representative gating strategy for identifying tumor-

infiltrating cDC1 and cDC2 in human lung cancer tissues. Live CD45+ Lineage (CD3, CD19, CD20, CD56, and CD66b)− CD14− HLA-DR+ CD11c+ cells were selected as cDCs and further distinguished by CD1c and CD141 expression: cDC1 (CD141+ CD1c−) and cDC2 (CD1c+ CD141−). XCR1 expression was further assessed in the cDC subset. **D,** Multiplex immunofluorescence of the negative control showing nuclei (DAPI) with the isotype control only, in tumor (left) and normal (right) tissues. Scale bars, 50 µm. Corresponds to the staining in Fig. 2H-I. **E,** Schematic diagram outlining the chemotaxis assay employed to assess cDC1 mobilization. **F,** Representative flow cytometry plots gated on CD45+ cells showing cDC1 in the upper chamber and CD4+ TRMs in the lower chamber. **G,** UMAP visualization of dendritic cell subsets (cDC1, cDC2, cDC3/mDC, and pDC) identified by scRNA-seq analysis. **H,** Dot plot showing canonical marker gene expression across identified dendritic cell subsets. **I,** Heatmap depicting the total number of significant ligand–receptor interactions among T-cell subsets (CD4+, CD8+ T and NK/NKT cells), and dendritic cell subsets (cDC1, cDC2, cDC3/mDC, and pDC), derived from cell–cell communication analysis of tumor samples. The data in (B) are expressed as the mean ± SD. Statistical analyses were performed using one-way ANOVA with Tukey's post hoc test (B); ns, non-significant; *p < 0.05; ** p < 0.01; *** p < 0.001; **** p < 0.0001.

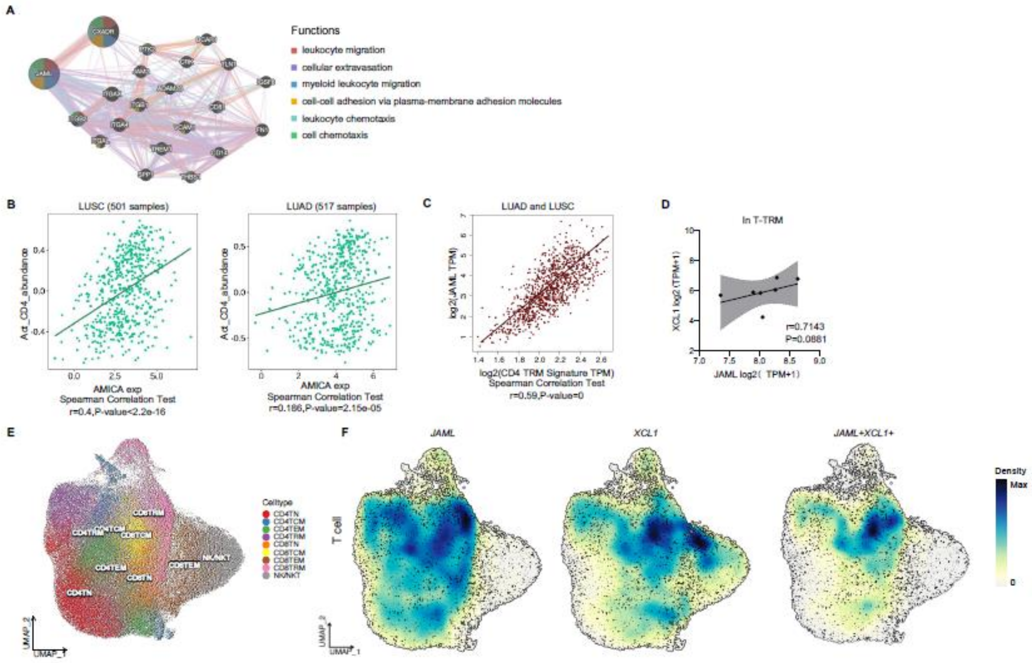

Extended Data Fig. 8 JAML signaling in tumor-infiltrating CD4+ TRMs

A, Human JAML protein interaction network and functional enrichment analysis generated via the GeneMANIA database (http://genemania.org). **B,** Correlations between JAML expression and immune infiltration in NSCLC (LUSC and LUAD) derived from the TISIDB platform (http://cis.hku.hk/TISIDB/index.php). **C,** Correlation scatter plots revealing the Spearman correlation between CD4+ TRM gene signatures and JAML expression in NSCLC samples from the TCGA database. TPM was used to quantify gene expression. r, Spearman correlation coefficient. **D,** Correlations between JAML and XCL1 expression in TDP, as assessed by Bulk RNA-seq (log_2TPM+1). **E,** UMAP visualization of single-cell transcriptomes showing NK/T-cell subsets in tumors, including CD4+ TN, TCM, TEM, and TRM; CD8+ TN, TCM, TEM, and TRM; and NK/NKT cells. **F,** UMAP visualization of the colocalization density of *JAML, XCL1,* and *JAML+XCL1+* across all T cells in tumors.

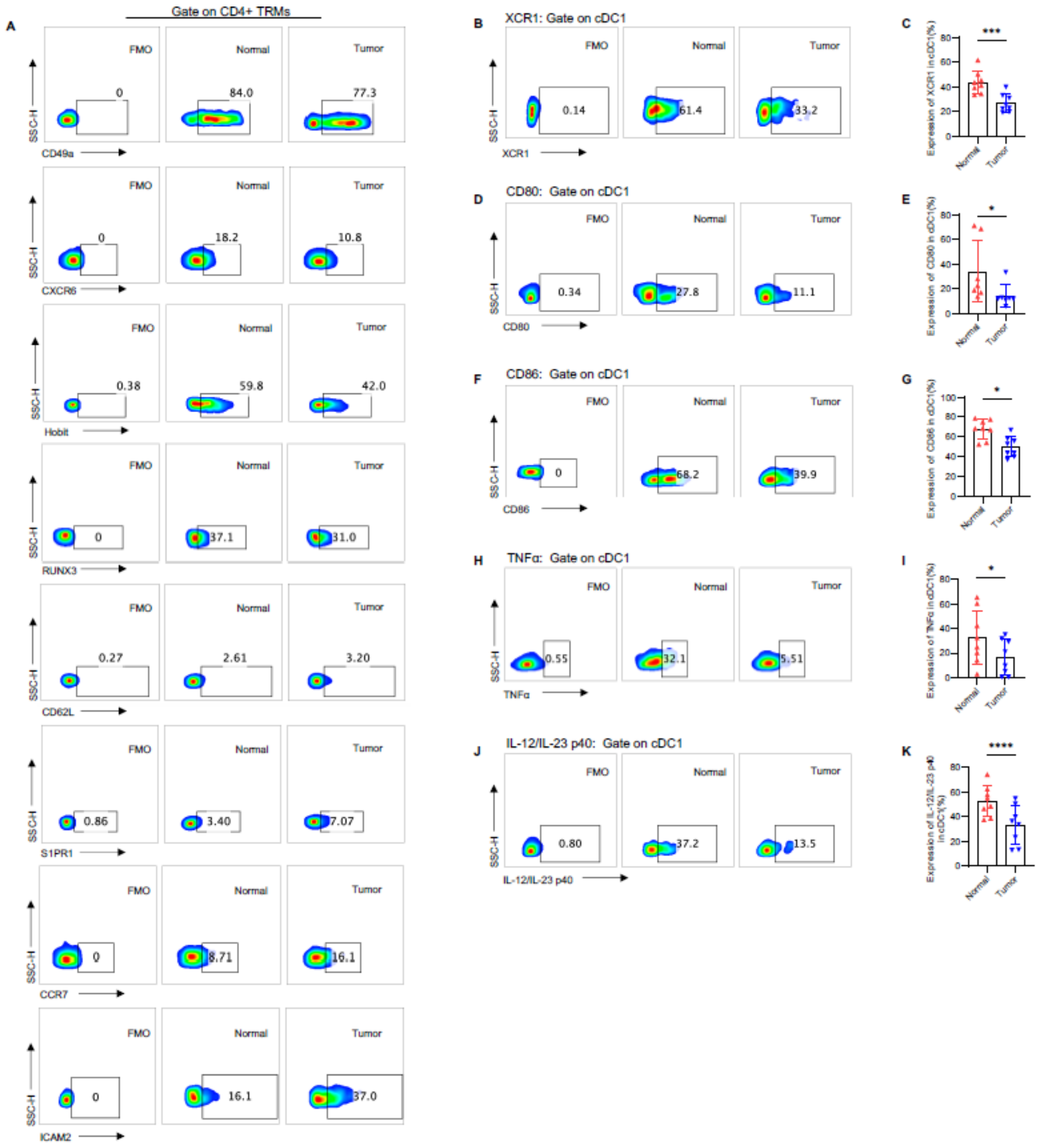

Extended Data Fig. 9 Dysregulated CD4+ TRMs and cDC1 signatures in tumors

A, Representative FCM plots of tissue-residency-related genes in CD4+ TRMs between normal and tumor tissues. The FMO control is shown on the left. **B-K,** FCM analysis of XCR1 (B-C), CD80 (D-E), CD86 (F-G), TNFα (H-I) and IL-12/IL-23 p40 (J-K) expression in cDC1 between normal and tumor tissues. Left, representative gating. Right, quantification. FMO controls are shown. The data in (C, E, G, I, and K) are expressed as the mean ± SD. Statistical analyses were

performed using unpaired Student’s t-tests (C, E, G, I, and K). ns, non-significant; *p < 0.05; **p < 0.01; ***p < 0.001; ****p < 0.0001.

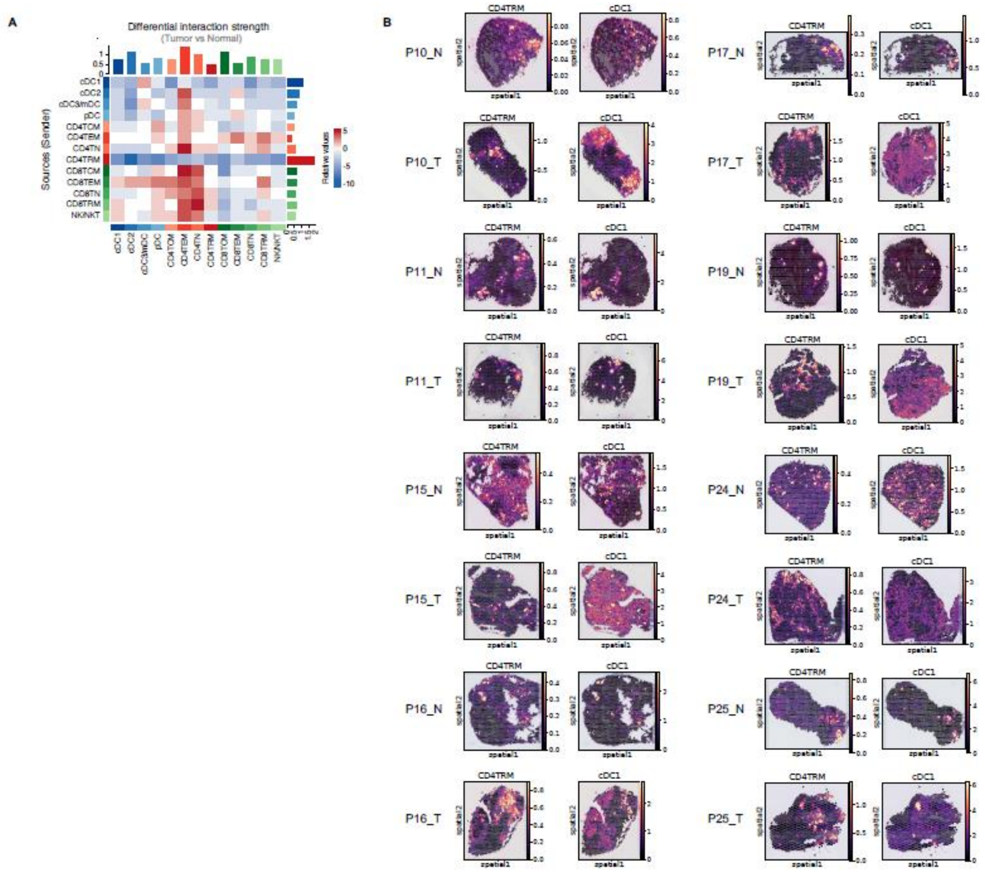

Extended Data Fig. 10 Reduced CD4+ TRM-cDC1 interactions and spatial proximity in tumors

A, Differential interaction strength between immune subsets in tumor versus normal tissues based on single-cell transcriptomic communication analysis. **B,** Spatial transcriptomics showing colocalization patterns of CD4+ TRMs and cDC1s across matched normal (N) and tumor (T) sections from individual patients (n=8).

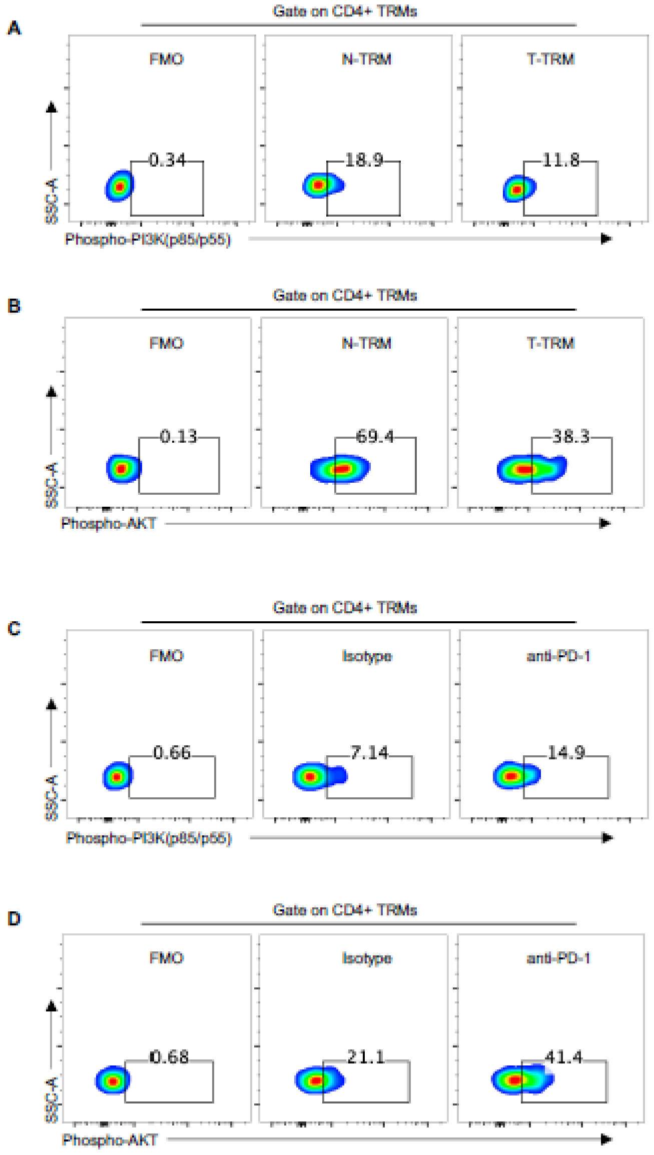

Extended Data Fig. 11 Anti-PD-1 treatment restores suppressed PI3K-AKT signaling in tumor-infiltrating CD4+ TRMs

A, Representative FCM plots of phospho-PI3K (A) and phospho-AKT (B) expression in CD4+ TRMs between normal and tumor tissues. **B,** Representative FCM plots of phospho-PI3K (C) and phospho-AKT (D) expression in CD4+ TRMs following isotype or anti-PD-1 antibody treatment.

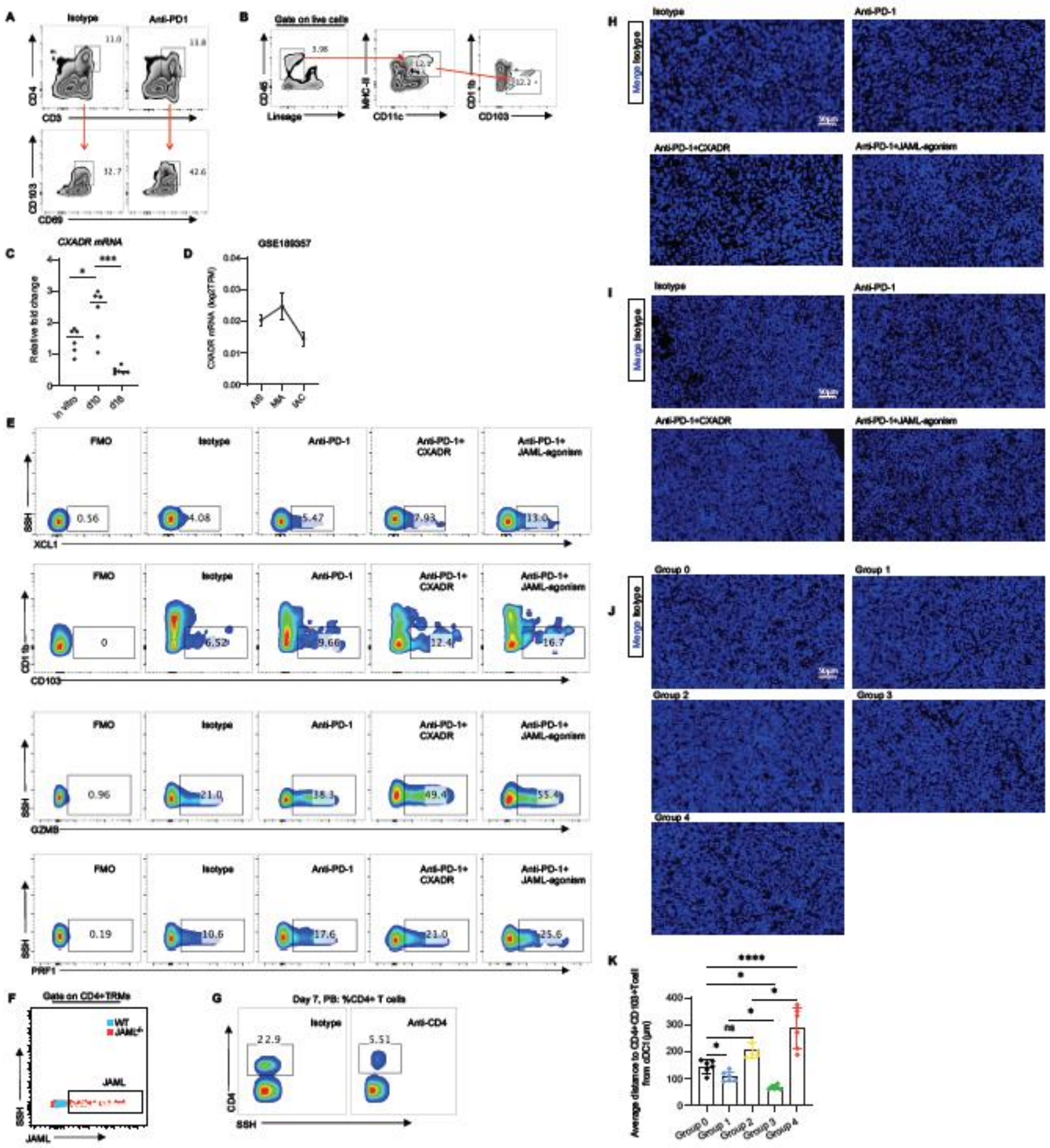

Extended Data Fig. 12 Combined anti-PD-1 and JAML agonist treatment enhances the anti-tumor function of CD4+ TRMs

A, FCM gating strategy for identifying CD4+ TRMs among CD4+ T cells in LLC tumors from the isotype and anti-PD-1-treated groups. **B,** Flow cytometry gating strategy for cDC1 in LLC tumors. Lineage (CD3ε, CD45R/B220, Ly6G). **C,** qPCR analysis of *CXADR* mRNA expression in LLC tumor tissues at Days 10 and 16 post-tumor challenge. n=6. **D,** *CXADR* mRNA expression of different stages of lung adenocarcinoma from GEO dataset (GSE189357); AIS (n = 3), MIA (n = 3), and IAC (n = 3). **E,** Representative FCM plots of XCL1, CD103,

GZMB, and PRF1 expression in CD4+ TRMs under isotype, anti-PD-1, anti-PD-1+CXADR, and anti-PD-1+JAML agonist treatment conditions. FMO controls are shown. **F,** Representative FCM plots gated on CD4+ TRMs showing JAML expression in CD4+ TRMs isolated from WT (blue) and JAML$^{-/-}$ (red) mice. **G,** Representative FCM plots showing the percentage of CD4+ T cells gated on CD3+ T cells in peripheral blood on Day 7 after isotype control or anti-CD4 antibody treatment. **H,** Multiplex immunofluorescence of the negative control showing nuclei (DAPI) with isotype control only, in tumors treated with isotype, anti-PD-1, anti-PD-1+CXADR, or anti-PD-1+JAML-agonism antibodies. Scale bars, 50 μm. Corresponds to the staining in Fig. 6C. **I,** Multiplex immunofluorescence negative control showing nuclei (DAPI) with isotype control only, in tumors treated with isotype, anti-PD-1, anti-PD-1+CXADR, or anti-PD-1+JAML-agonism. Scale bars, 50 μm. Corresponds to the staining in Fig. 6F. **J,** Multiplex immunofluorescence of the negative control showing nuclei (DAPI) with isotype control only, in tumors from Groups 0–4. Scale bars, 50 μm. Corresponds to the staining in Fig. 6T. **K,** Average distance from cDC1 to the nearest CD4+CD103+ T cells (μm) in CMT-167 tumors across Groups 0–4. The data in (K) are expressed as mean ± SD. Statistical analyses were performed using one-way ANOVA with Tukey's post hoc test (C, K). ns, non-significant; *$p < 0.05$; **$p < 0.01$; ***$p < 0.001$; ****$p < 0.0001$.

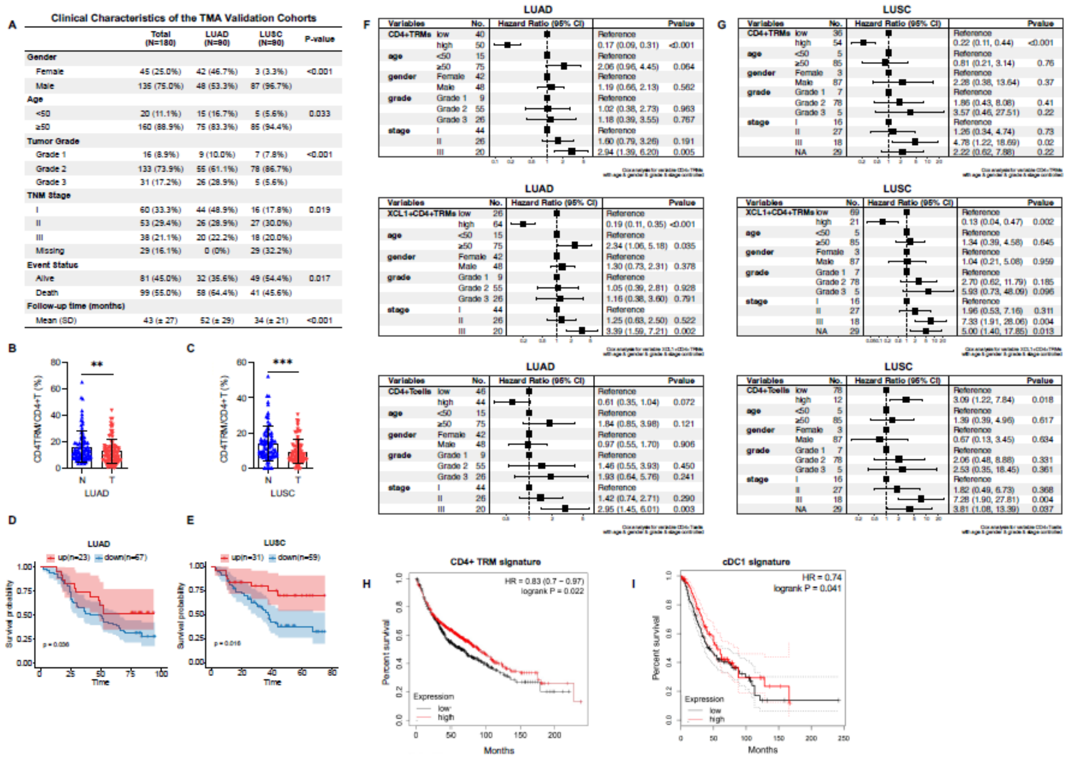

Extended Data Fig. 13 Prognostic significance of CD4+ TRMs in LUAD and LUSC

A, Clinical characteristics of the TMA validation cohorts. Summary table showing patient demographics and clinical parameters for LUAD (n=90) and LUSC (n=90) cohorts, including gender, age, tumor grade, TNM stage, event-free survival status, and follow-up time. **B-C,** Frequencies of CD4+ CD103+ T-cells among CD4+ T cells in paired normal (N) and tumor (T) tissues from lung adenocarcinoma (LUAD, B) and lung squamous cell carcinoma (LUSC, C). **D-E,** Kaplan–Meier overall survival (OS) curves stratified by the change in CD4+CD103+ T cell abundance in tumors compared with that in paired normal tissues in LUAD (D, n = 90) and LUSC (E, n = 90). Patients were classified as up or down on the basis of the change in the percentage of CD4+CD103+ T cells among CD4+ T cells, and survival differences were assessed by the log-rank test. **F-G,** Multivariate Cox regression analyses of prognostic factors in LUAD (**F**) and LUSC (**G**). Forest plots for overall survival showing hazard ratios (HRs) with 95% CIs for CD4+ TRMs (top), XCL1+CD4+ TRMs (middle), and

total CD4+ T cells (bottom). Patients were stratified into high and low groups on the basis of the optimal cutoff values. Models were adjusted for age, gender, tumor grade, and TNM stage. Numbers indicate sample sizes within each category. **H-I,** Kaplan–Meier overall survival curves stratified by the expression of the CD4+ TRM signature (*CD4+ ITGAE+ CXCR6 +ZNF683+*) (H) and cDC1 signature (*XCR1+ CLNK+ BATF3+ CLEC9A+*) (I) in human NSCLC samples from the TCGA database. P values were calculated using the log-rank test. The figure H was created using the Kaplan–Meier Plotter (www.kmplot.com), and I was created using the Gene Expression Profiling Interactive Analysis (GEPIA) (http://gepia.cancer-pku.cn/). The data in (B, C) are expressed as the mean ± SD. Statistical analyses were performed using paired Student's t-tests (B, C). ns, non-significant; *p < 0.05; **p < 0.01; ***p < 0.001; ****p < 0.0001.

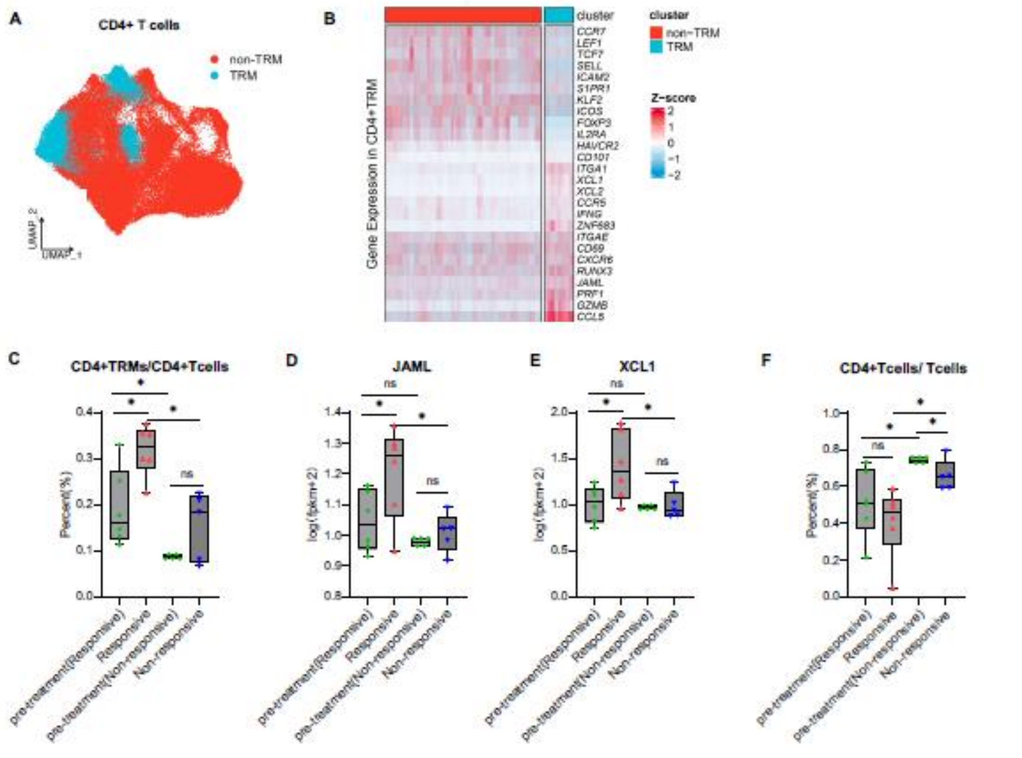

Extended Data Fig. 14 ***Tumor-infiltrating CD4+ TRMs correlate with immunotherapy efficacy in NSCLC***

A, UMAP embedding of CD4+ T cells from NSCLC tumor tissues (GSE179994), with TRM cells (blue) and non-TRM cells (red) identified by scRNA-seq. Each dot represents a single cell. **B,** Heatmap showing the normalized expression of representative tissue-residency-associated genes in TRM compared with non-TRM. **C-F**, Proportions of CD4+ TRMs among total CD4+ T cells (**C**), log2(fpkm+2) expression of JAML (**D**) and XCL1 (**E**), and proportions of CD4+ T cells among total T cells (**F**) in paired pre- and post-treatment samples from responsive and non-responsive patients. The data in (C-F) are expressed as the mean ± SD. Statistical analyses were performed using one-way ANOVA with Tukey's post hoc test (C-F). ns, non-significant; *p < 0.05; **p < 0.01; ***p < 0.001; ****p < 0.0001.

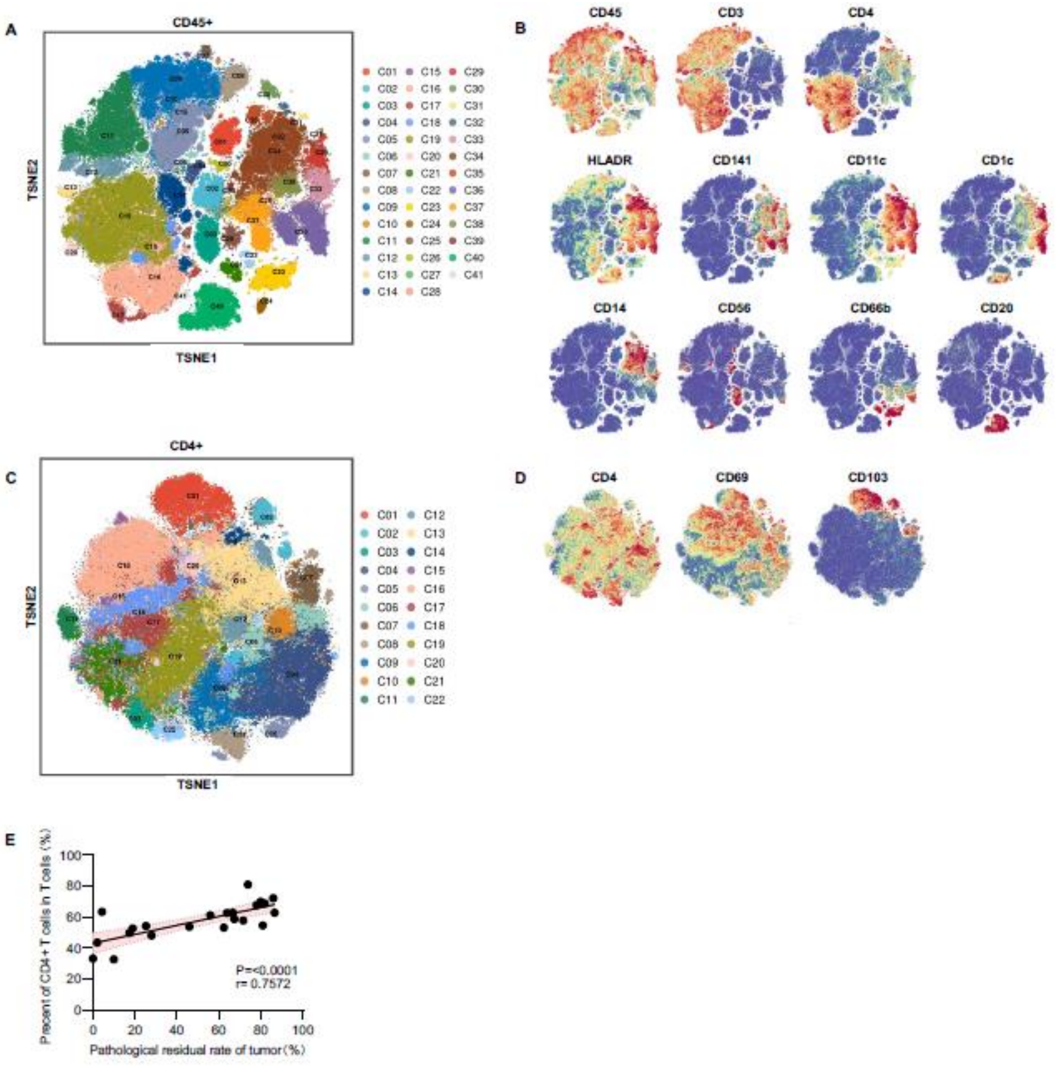

Extended Data Fig. 15 The CyTOF landscape of CD4+ TRMs and cDC1

A-B, T-SNE plots showing the (A) embedding of CD45+ cells from NSCLC tumors post-anti-PD-1 therapy (n=23), analyzed via CyTOF using a PhenoGraph clustering algorithm. T-SNE plots (B) displaying the overlaid signal intensity of selected phenotypic markers in CD45+ cells. Red dashed lines outling cell subpopulations of CD4+ T cells (Clusters 13, 14, 16, 17, 18, 19, and 20), cDCs (Clusters 27, 28, 29, and 38), cDC1 (Clusters 28 and 38). Phenotype: CD4+ T cells (CD45+ CD3+ CD4+), cDCs: (CD45+ CD3- CD20- CD14- CD56- CD66b-HLADR+ CD11c+), cDC1 (CD45+ CD3- CD20- CD14- CD56- CD66b- HLADR+ CD11c+ CD141+). **C-D,** T-SNE plots embedding of CD4+ T cells and displaying the overlaid signal intensity of selected phenotypic markers (CD4+ CD69+ CD103+) in CD4+ T cells. **E,** Correlation analyses showing the relationship between CD4+ T cells within T cells and the residual pathological tumor rate following anti-PD-1 immunotherapy (n=23). Spearman correlation coefficients (r) and P values are indicated.
